# Consequences of a Diagnostic Label: A Systematic Scoping Review and Thematic Framework

**DOI:** 10.3389/fpubh.2021.725877

**Published:** 2021-12-22

**Authors:** Rebecca Sims, Zoe A. Michaleff, Paul Glasziou, Rae Thomas

**Affiliations:** Institute for Evidence-Based Healthcare (IEBH), Health Science and Medicine, Bond University, Gold Coast, QLD, Australia

**Keywords:** labelling, diagnosis, consequences, qualitative, scoping review

## Abstract

**Objectives:** To develop a thematic framework for the range of consequences arising from a diagnostic label from an individual, family/caregiver, healthcare professional, and community perspective.

**Design:** Systematic scoping review of qualitative studies.

**Search Strategy:** We searched PubMed, Embase, PsycINFO, Cochrane, and CINAHL for primary studies and syntheses of primary studies that explore the consequences of labelling non-cancer diagnoses. Reference lists of included studies were screened, and forward citation searches undertaken.

**Study Selection:** We included peer reviewed publications describing the perceived consequences for individuals labelled with a non-cancer diagnostic label from four perspectives: that of the individual, their family/caregiver, healthcare professional and/or community members. We excluded studies using hypothetical scenarios.

**Data Extraction and Synthesis:** Data extraction used a three-staged process: one third was used to develop a preliminary framework, the next third for framework validation, and the final third coded if thematic saturation was not achieved. Author themes and supporting quotes were extracted, and analysed from the perspective of individual, family/caregiver, healthcare professional, or community member.

**Results:** After deduplication, searches identified 7,379 unique articles. Following screening, 146 articles, consisting of 128 primary studies and 18 reviews, were included. The developed framework consisted of five overarching themes relevant to the four perspectives: *psychosocial impact* (e.g., positive/negative psychological impact, social- and self-identity, stigma), *support* (e.g., increased, decreased, relationship changes, professional interactions), *future planning* (e.g., action and uncertainty), *behaviour* (e.g., beneficial or detrimental modifications), and *treatment expectations (e.g., positive/negative experiences)*. Perspectives of individuals were most frequently reported.

**Conclusions:** This review developed and validated a framework of five domains of consequences following diagnostic labelling. Further research is required to test the external validity and acceptability of the framework for individuals and their family/caregiver, healthcare professionals, and community.

## Introduction

Worldwide there has been an increase in the use of diagnostic labels for both physical and psychological diagnoses (1, 2). Diagnoses reflects the process of classifying an individual who presents with certain signs and symptoms as having, or not having, a particular disease (3). The diagnostic process can involve various assessments and tests, however, culminates to a “diagnostic label” that is communicated to the individual (4). The term “diagnostic label” will be used to indicate diagnosis or labelling of health conditions listed in current diagnostic manuals (5, 6). Diagnostic definitions and criteria continue to expand and, with this, individuals who are asymptomatic or experience mild symptoms are increasingly likely to receive a diagnostic label (7, 8). It is acknowledged that the consequences of a diagnostic label are likely individual, and how each is perceived is dependent on numerous internal (e.g., medical history, age, sex, culture) and external (e.g., service availability, country) factors, and differs by perspective (9). Motivation for expanding disease definitions and increased labelling includes the presumed benefits such as validation of health concerns, access to interventions, and increased support (3, 10). However, often less considered are the problematic or negative consequences of a diagnostic label. This may include increased psychological distress, preference for invasive treatments, greater sick role behaviour, and restriction of independence (11–14). Additionally, research indicates the impact of a label is diverse and varies depending on your perspective as an individual labelled (15, 16), family/caregiver (15, 17, 18), or healthcare professional (15, 19).

Psychosocial theories, including social constructionism, labelling theory, and modified labelling theory, have attempted to explain the varied influence of labels on an individuals' well-being and identity formation, in addition to society's role in perpetuating assumptions and necessity of particular labels (3, 20–22). In terms of quantifying this impact, research to date has examined the impact of changes to diagnostic criteria (e.g., cut-points/thresholds), how and when diagnoses are provided (e.g., tests used, detection through screening, or symptom investigation), the prevalence of diagnoses, or treatment methods and outcomes (4, 23–26). However, clinicians and researchers have paid relatively less attention to the consequences a diagnostic label has on psychological well-being, access to services, and perceived health. Of particular concern, are the implications of a diagnostic label for people who are asymptomatic or present with mild signs and symptoms are of critical importance as it is this group of people who are less likely to benefit from treatments and are at greater risk of harm (4, 27).

The limited work in this area has reported on individual diagnostic labels, used hypothetical case scenarios, or failed to differentiate between condition symptoms and condition label (28, 29). Few studies have synthesised the real-world consequences of diagnostic labelling, with existing syntheses restricted to a specific condition or limited in the methodological approach used (e.g., hypothetical case-studies) (30–32). This suggests a paucity of information available for individuals, their family/caregivers, healthcare professionals, and community members to understand the potential consequences of being given a diagnostic label. Therefore, the aim of this scoping review is to identify and synthesise the potential consequences of a diagnostic label from the perspective of an individual who is labelled, their family/caregiver, healthcare professional, and community members.

## Methods

### Design

This systematic scoping review was conducted and reported in accordance with the published protocol (33), the Joanna Briggs Methodology for Scoping Reviews (34), and Preferred Reporting Items for Systematic Reviews and Meta-Analyses Extension for Scoping Reviews (PRISMA-ScR) (35). Originally, we proposed to report the results of both qualitative and quantitative studies together, however, due to the large volume of included studies and the richness of the data, only results from the qualitative studies are reported in this paper. Results from quantitative studies will be reported separately. Subsequently, this article presents the results of the qualitative synthesis.

### Search Strategy

An electronic database search was conducted in PubMed, Embase, PsycINFO, Cochrane, and CINAHL from database inception to 8 June 2020. The search strategy combined medical subject headings and key word terms related to “diagnosis” and “effect” (see PubMed Search Strategy in Supplementary Material). Forward and backward citation searching was conducted to identify additional studies not found by the database search.

### Inclusion Criteria

We included peer reviewed publications, both primary studies and systematic or literature reviews, that reported on consequences of a diagnostic label for a non-cancer diagnosis. Included studies could report consequences from the perspectives of the individual, their family, friends, and/or caregivers, healthcare professional, or community member.

Studies reporting labelling of cancer conditions were excluded as existing research suggests that individuals labelled as having a cancer condition may report different experiences, for example, associating the condition with lethality, or desiring invasive treatments, to those labelled with other physical (e.g., diabetes, polycystic ovarian syndrome) or psychological (e.g., autism spectrum disorder, dementia) diagnoses (36–39). Similarly, hypothetical scenarios, or labelling of individuals with intellectual disabilities and/or attributes such as race, sexual identity, or sexual orientation were also excluded.

### Study Selection

Published studies retrieved by database searches were exported to EndNote and deduplicated. Two reviewers (RS, LK) independently screened ~10% of studies and achieved an interrater reliability of kappa 0.92. Disagreements were resolved by discussion or additional reviewers (RT, ZAM) as necessary. The remaining screening was completed by one reviewer (RS), with studies identified as unclear for inclusion reviewed by additional reviewers (RT, ZAM) as required.

### Preliminary Framework Development

Prior to commencement of this scoping review, a poll was conducted on social media (Twitter, Facebook) asking a single question about people's experiences of receiving a diagnostic label and any associated consequences. A preliminary framework was developed and agreed upon by members of the research team from the responses received from 46 people. The preliminary framework included five primary themes and seven sub-themes detailed in the published protocol (33). This preliminary framework was used as a starting point from which to iteratively develop and synthesise the range of consequences that emerged from the studies included in this review.

### Data Extraction and Analysis

Once eligible articles were identified, data was extracted and analysed from randomly selected articles using a three-stage process. The first stage (i.e., first third of randomly selected articles) was used to iteratively develop the framework. The second stage (i.e., second third of randomly selected articles) was used to examine the framework for completeness and explore the extracted data for thematic saturation. The final third of included studies was to be extracted and analysed only if saturation had not occurred. Thematic saturation was defined as the non-emergence of new themes that would result in revision of the framework (40).

Three authors (RS, RT, and ZAM) independently extracted data from 10% of the first third of included studies and mapped this to the preliminary framework. As new consequences were identified the framework was revised and subthemes emerged. Conflicts were resolved through discussion. One reviewer (RS) completed extraction of the remaining studies in the first third. Reflexivity was achieved through regular discussions with an additional reviewer (RT or ZAM) to ensure articles were relevant, coding was reliable, and homogeneity existed between data extracted to major themes and subthemes (41, 42). When data extraction was completed, two additional reviewers (RT and ZAM) examined the extracted data and disagreements in coding were resolved through discussion.

Extracted data included study characteristics (author, journal, year of publication, study country, and setting), participant characteristics (number of participants, age, diagnostic label), and abstracted themes and relevant supporting quotes identified by the authors of the included studies that pertained to the consequences of a diagnostic label. Direct quotes were not extracted in isolation to preserve the author's meaning and ensure contextual understanding from the primary study was retained. These qualitative meta-analysis techniques have been described elsewhere (43–45).

## Results

### Search Results

**Figure 1:**
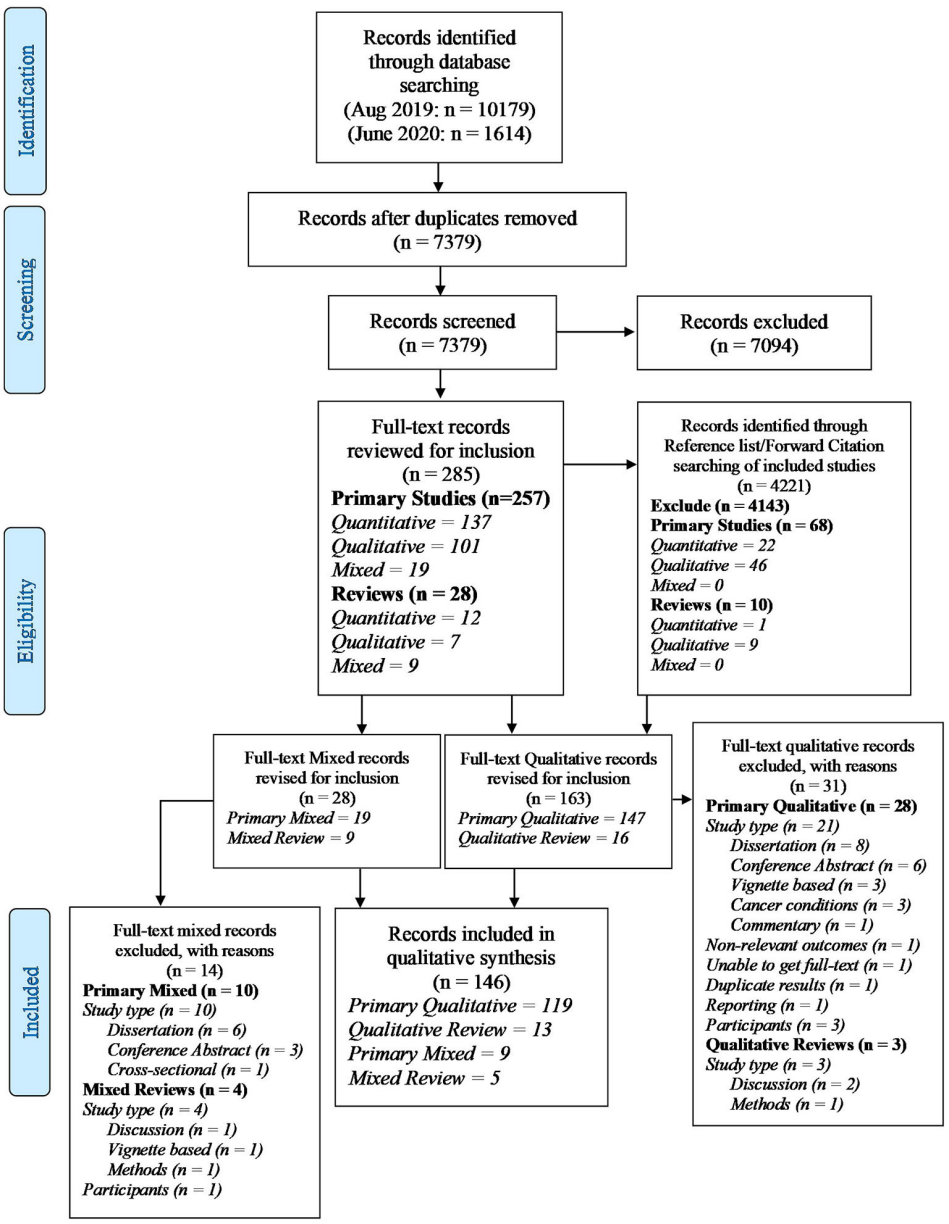
PRISMA-ScR flow diagram.

Searches identified 16,014 unique records which we screened for inclusion. Full texts were retrieved for 191 qualitative studies, of which 146 (128 studies, 18 reviews) were included in this systematic scoping review (Figure 1). Data extraction was completed using the staged processed described above. Saturation of themes was achieved by the conclusion of the second stage of data extraction. Therefore, 97 studies (of which 13 were reviews) directly informed our results.

Of the studies that directly informed the coding framework, 61 examined physical diagnostic labels (e.g., diabetes, female reproductive disorders) and 36 examined psychological diagnostic labels (e.g., autism spectrum disorder, dementia). Over half of the studies (58%, 56/97) reported individual perspectives on being labelled with a diagnostic label, 9% (9/97) reported on family/caregiver perspectives, 14% (14/97) reported healthcare professional perspectives, and 19% (18/97) reported multiple (including community) perspectives. Key characteristics of the included studies are provided in Table 1.

**Table 1:** Key characteristics of extracted qualitative studies and reviews.

**References**	Condition* (Scr, Sym, NR, Mix)	**Country**	**Participants**	***N***	**Age Range (years)**	**% Female**	**Data collection**	**Data analysis**
**Cardiovascular disease**								
Asif et al. (46)	Cardiac conditions (Scr)	USA	Individual	25	14–35	48	Individual semi-structured interview	Consensual qualitative research
**Chronic kidney disease**								
Daker-White et al. (47)	Chronic kidney disease (Sym)	UK	Individual (control arm of trial)	13	59–89	69.2	Individual interview	Grounded theory
			Individual (intervention arm of trial)	13	59–89	61.5		
**Diabetes**								
Twohig et al. (48)	Pre-diabetes (Sym)	UK	Individual	23	37–81	56	Individual semi-structured interview	Thematic analysis with interpretivist analytical approach
Burch et al. (49)	Pre-diabetes (NR)	UK	GP, GP registrar, nurse practitioners, practise nurse, healthcare assistant, patient advocates	17	NR	NR	Individual semi-structured interview	Grounded theory approach
				7	NR	NR	Focus groups (*n* = 2)	
de Oliveira et al. (50)	Diabetes (NR)	Brazil	Individual	16	NR	NR	Focus groups (*n* = 4)	Thematic content analysis
Due-Christensen et al. (51)	Type 1 diabetes (NR)	Canada, Sweden, UK	Individual	124	23–58	NR	Systematic review	Meta-synthesis
Sato et al. (52)	Type 1 diabetes (NR)	Japan	Individual	13	21–35	77	Individual semi-structured interview	NR
Jackson et al. (53)	Type 1 diabetes (Sym)	UK	Siblings	41	7–16	58.5	Individual semi-structured interview	Grounded theory
Fharm et al. (54)	Type 2 diabetes (NR)	Sweden	GPs	14	43–64	57.1	Focus group (*n* = 4)	Qualitative content analysis
Kaptein et al. (55)	GDM (Scr)	Canada	Individual	19	29–50	100	Semi-structured interview	Conventional content analysis
Singh et al. (56)	GDM (Scr)	USA	Individual	29	NR	100	Semi-structured interview	Thematic analysis
**Female reproduction**								
Copp et al. (57)	PCOS (Sym)	Australia	Individual	26	18–45	100	Individual semi-structured interview	Framework
Copp et al. (58)	PCOS (Sym)	Australia	GPs, gynaecologists, endocrinologists	36	NR	72.2	Individual semi-structured interview	Framework analysis
Newton et al. (59)	Pelvic inflammatory disease (NR)	Australia	Individual	23	18–46	100	Semi-structured interview	Inductive thematic approach
O'Brien et al. (60)	Anti-Mullerian hormone testing (Scr)	Ireland	Individual	10	24–69	100	Semi-structured interview	Thematic analysis
Patterson et al. (61)	MRKH (Sym)	UK	Individual	5	18–22	100	Individual semi-structured interview	Interpretative phenomenological approach
Harris et al. (62)	Pre-eclampsia (Scr)	UK	Individual	10	28–36	100	Semi-structured interview	Framework analysis
**Genome/Chromosome**								
Delaporte (63)	Facioscapulohumeral dystrophy (Sym)	France	Individual	22	NR	NR	Individual semi-structured interview	Content analysis
			Neurologists	10	NR	NR		
Houdayer et al. (64)	Chromosomal abnormalities (Scr)	France	Parents	60	NR	63.3	Individual semi-structured interview	Transversal analysis
			Geneticists	5	NR	NR		
**HIV/AIDS**								
McGrath et al. (65)	AIDS (NR)	Uganda	Individual	24	18–55	58	Individual semi-structured interview and observations	NR
			Family members	22	NR	NR		
Anderson et al. (66)	HIV (NR)	UK	Individual	25	NR	20	Individual semi-structured interview	NR
Freeman (67)	HIV (NR)	Malawi	Individual	18	50–70	NR	Individual interview	Constructivist grounded theory
			Individual attending support group	NR	30–75	NR	Focus group (*n* = 3)	
Kako et al. (68)	HIV (NR)	Kenya	Individual	40	26–54	100	Individual interview	Multistage narrative analysis
Kako et al. (69)	HIV (NR)	Kenya	Individual	24	20–39	100	Semi-structured interview	Thematic analysis
Stevens and Hildebrandt (70)	HIV (NR)	USA	Individual	55	23–54	100	Individual interview	NR
Firn and Norman (71)	HIV/AIDS (Sym)	UK	Individual	7	NR	28.6	Individual semi-structured interview	Inductive categorisation
			Nurses	10	NR	80		
**Immune system**								
Hale et al. (72)	Systemic lupus erythematosus (Sym)	UK	Individual	10	26–68	100	Individual semi-structured interview	Interpretative phenomenological approach
**Infectious/Parasitic**								
Almeida et al. (73)	Leprosy (NR)	Brazil	Individual	14	21–80	57	Individual semi-structured interview	NR
Silveira et al. (74)	Leprosy (NR)	Brazil	Individual	5	36–70	NR	Unstructured interview	Content analysis
Zuniga et al. (75)	Tuberculosis (NR)	USA	Individual	13	NR	0	Semi-structured interview	Secondary analysis using qualitative descriptive methods
Dodor et al. (76)	Tuberculosis (NR)	Ghana	Individual	34	NR	29.4	Individual semi-structured interview	Grounded theory
				65	NR	24.6	Focus groups (*n* = 6)	
			Community members	66	NR	56.1	Individual semi-structured interview	
				177	NR	46.3	Focus groups (*n* = 16)	
**Metabolic**								
Bouwman et al. (77)	Fabry disease (NR)	Netherlands	Individual	30	12–68	57	Semi-structured interview	NR
**Musculoskeletal**								
Erskine et al. (78)	Psoriatic arthritis (Sym)	UK	Individual	41	46.6–69–4	51.2	Focus groups (*n* = 8)	Secondary analysis using deductive thematic analysis
Martindale and Goodacre (79)	Axial spondyloarthritis (Sym)	UK	Individual	10	26–49	30	Individual semi-structured interview	Interpretative phenomenological approach
Hopayian and Notley (80)	Low back pain/sciatica (Sym)	Australia, Finland Ireland, Israel, Netherlands, Norway, UK, USA	Individual	NR	NR	NR	Systematic review	Thematic content analysis
Barker et al. (81)	Osteoporosis (Mix)	Brazil, Canada, Denmark, Sweden, UK, USA	Individual	773	33–93	89.2	Review	Meta-ethnography
Hansen et al. (82)	Osteoporosis (NR)	Denmark	Individual	15	65–79	100	Individual interview	Phenomenological hermeneutic approach
Weston et al. (83)	Osteoporosis (Scr)	UK	Individual	10	68–79	100	Individual semi-structured interview	Interpretative phenomenological approach
Boulton (84)	Fibromyalgia (Sym)	Canada, UK	Individual	31	21–69	81	Individual semi-structured interview	Narrative analysis
Madden Sim (85)	Fibromyalgia (Sym)	UK	Individual	17	25–55	94	Individual semi-structured interview	Induction-abduction method
Mengshoel et al. (86)	Fibromyalgia (Sym)	Africa, Belgium, Canada, Finland, France, Japan, Mexico, Norway, South Africa, Spain, Sweden, UK, USA	Individual	475	16–80	94.7	Review	Meta-ethnography
Raymond and Brown (87)	Fibromyalgia (Sym)	Canada	Individual	7	38–47	85.7	Individual semi-structured interview	Phenomenological approach
Sim Madden (88)	Fibromyalgia (Sym)	Canada, Norway, Sweden, UK, USA	Individual	383	NR	94	Review	Meta-synthesis
Undeland and Malterud (89)	Fibromyalgia (Sym)	Norway	Individual	11	42–67	100	Focus Groups (n = 2)	Systematic text condensation
**Nervous system**								
Chew-Graham et al. (90)	CFS/ME (Sym)	UK	GPs	22	NR	NR	Individual semi-structured interview	Thematic analysis
Hannon et al. (91)	CFS/ME (Sym)	UK	Individual	16	28–64	68.8	Individual semi-structured interview	hematic analysis using modified grounded theory
			Carers	10	46–71	50		
			GPs, specialists, practise nurses	18	NR	77.8		
De Silva et al. (92)	CFS/ME (Sym)	UK	Individual	11	NR	72.7	Individual semi-structured interview	Secondary analysis
			Carers	2	NR	50		
			GPs	9	NR	67		
			Community Leaders	5	NR	40		
Johnston et al. (93)	MND (Sym)	UK	Individual	50	38–85	34	Individual interview	NR
Zarotti et al. (94)	MND (Sym)	UK	Dietitians, dietetics managers, MND specialist nurses, Speech and language therapists, MND coordinators, service user representatives, GPs, physiotherapists	51	NR	90	Focus Group (*n* = 5)	Thematic analysis
Johnson (95)	Multiple sclerosis (Sym)	UK	Individual	24	34–67	58.3	Individual interview	Framework of data reduction, data display, and conclusion drawing/verification
Thompson et al. (96)	Non-epileptic seizures (Sym)	UK	Individual	8	NR	100	Semi-structured interview	Interpretative phenomenological approach
Wyatt et al. (97)	Non-epileptic attack disorder (Sym)	UK	Individual	6	29–55	83.3	Semi-structured interview	Descriptive phenomenological approach using inductive analytic approach
			Partners	3	NR	0		
**Neurological**								
Nochi (98)	Traumatic brain injury (Sym)	USA	Individual	10	24–54	20	Semi-structured interview	Grounded theory
				13	26–61	61.5	Written narrative accounts	
Daker-White et al. (99)	Ataxia (Sym)	NR	Individual	NR	NR	NR	Review of internet discussion forums	NR
			Partners or parents	NR	NR	NR		
**Newborn/Foetal**								
Hallberg et al. (100)	22q11 Deletion syndrome (Scr)	Sweden	Parents	12	NR	83.3	Conversational interview	Classical grounded theory
Johnson et al. (101)	Cystic fibrosis (Scr)	UK	Parents	8	NR	62.5	Semi-structured interview	Interpretative phenomenological analysis
Dahlen et al. (102)	GERD (Sym)	Australia	Child health nurses; enrolled/mothercraft nurses; psychiatrists; GPs; paediatricians	45	NR	NR	Focus Group (*n* = 8)	Thematic analysis
**Sleep-Wake disorder**								
Zarhin (103)	Obstructive sleep apnoea (Sym)	Israel	Individual	65	30–66	47.7	Interview	Coded thematically and analysed based on constructivist grounded theory
**Sexually transmitted**								
Mills et al. (104)	Chlamydia trachomatis (Scr)	UK	Individual	25	18–28	68	Individual semi-structured interview	Inductive
Rodriguez et al. (105)	HPV (NR)	Australia, Brazil, Canada, Colombia, Denmark, Ireland, Mexico, Peru, Sweden, UK, USA	Individual	34	NR	85.3	Scoping review	NR
**Multiple physical diagnoses**								
Kralik et al. (106)	Adult-Onset chronic illness (Sym)	Australia	Individual	81	NR	100	Written narrative accounts	Secondary analysis
	Diabetes (Sym)		Individual	10	NR	100	Focus groups (*n* = 8)	Secondary analysis
**Bipolar disorder**								
Fernandes et al. (107)	Bipolar disorder (Sym)	Australia	Individual	10	29–68	100	Individual semi-structured interview	Constant comparative method
Proudfoot et al. (108)	Bipolar disorder (Sym)	Australia	Individual	26	18–59	54	Online communication with public health service	Phenomenology and lived experience framework
**Depression**								
Wisdom and Green (109)	Depression (Sym)	USA	Individual	15	NR	53.3	Individual semi-structured interview	Modified grounded theory
Chew-Graham et al. (110)	Depression (Sym)	UK	Inner-city GPs	22	NR	NR	Individual semi-structured interview	Inductive thematic analysis
			Semi-rural/Suburban GPs	13	NR	NR		
**Neurocognitive**								
Beard and Fox (111)	AD; MCI (Sym)	USA	Individual	8	NR	NR	Individual semi-structured interview	Grounded theory
				32	NR	NR	Focus group (*n* = 6)	
Bamford et al. (112)	Dementia (Sym)	Australia, Canada, Ireland, Italy, Netherlands, Scotland, Sweden, UK, USA	Individual	NR	NR	NR	Systematic review	NR
			Carers	NR	NR	NR		
			GPs, Psychiatrists, Psychologists, Geriatricians, Nurses, Neurologists	NR	NR	NR		
Bunn et al. (113)	Dementia; MCI (Sym)	Asia, Australia, Canada, Europe, New Zealand, UK, USA	Individual	74	40–97	NR	Review	Thematic synthesis
			Carers	72	40–97	NR		
Robinson et al. (114)	AD; Dementia (Sym)	UK	Individual	9	73–85	55.6	Semi-structured interview with partner	Interpretative phenomenological analysis
			Partners	9	68–81	NR		
Ducharme et al. (115)	AD (Sym)	Canada	Spouses	12	48.1–61.9	66.7	Individual semi-structured interview	Phenomenology
Abe et al. (116)	Dementia (Sym)	Japan	Rural GPs	12	NR	25	Individual semi-structured interview	Thematic analysis
			Urban GPs	12	NR	33		
Phillips et al. (117)	Dementia (Sym)	Australia	GPs	45	NR	NR	Individual semi-structured interview	Thematic analysis
Walmsley and McCormack (118)	Dementia (Sym)	Australia	Aged Care directors; GP, nurse unit manager, dementia body representative	8	48–60	75	Individual semi-structured interview	Interpretative phenomenological analysis
Werner and Doron (119)	AD (Sym)	Israel	Social workers	16	NR	NR	Focus group (*n* = 3)	Thematic analysis using constant comparative method
			Lawyers	16	NR	NR		
**Neurodevelopmental**								
Carr-Fanning and Mc Guckin (120)	ADHD (Sym)	Ireland	Individual	15	7–18	40	Individual semi-structured interview	Thematic analysis
			Parents	17	NR	88.2		
Mogensen and Mason (121)	ASD (Sym)	Australia	Individual	5	13–18	40	Individual interview, communication cards, e-mails	Interpretative phenomenological analysis
Fleischmann (122)	ASD (Sym)	NR	Parents	33	NR	NR	Web page mining	Grounded theory
Hildalgo et al. (123)	ASD (Sym)	USA	Primary caregiver	46	NR	100	Individual structured interview	Thematic analysis
Loukisas and Papoudi (124)	ASD (Sym)	Greece	Parent	5	35–45	100	Review of written blogs	Content analysis
Selman et al. (125)	ASD (Sym)	UK	Parent	15	28–56	0	Individual semi-structured interview	Thematic analysis
Smith et al. (126)	ASD (Sym)	NR	Individual	14	8–21	NR	Systematic review	NR
			Parents	7	NR	NR		
**Obsessive compulsive disorder**								
Pedley et al. (127)	OCD (Sym)	UK	Family member	14	25–71	NR	Individual semi-structured interview	Thematic analysis
**Peri/Postnatal anxiety and/or depression**								
Ford et al. (128)	Perinatal anxiety and depression (Scr)	Australia, UK	GPs	405	NR	NR	Review	Meta-ethnography
Chew-Graham et al. (129)	Postnatal Depression (Sym)	UK	GPs	19	NR	NR	Individual semi-structured interview	Inductive thematic analysis
			Health Visitors	14	NR	NR		
**Personality disorder**								
Horn et al. (130)	BPD (Sym)	UK	Individual	5	23–44	80	Individual semi-structured interview	Interpretative phenomenological analysis
Lester et al. (131)	BPD (Sym)	NR	Individual	172	NR	75	Systematic review	Thematic analysis
Nehls (132)	BPD (Sym)	USA	Individual	30	NR	100	Individual semi-structured interview	Interpretative phenomenological analysis
**Schizophrenia/psychotic disorder**								
Thomas et al. (133)	Schizophrenia (Sym)	NR	Individual	97	NR	NR	Online survey	Thematic analysis
Welsh and Tiffin (134)	At risk mental state (Sym)	UK	Individual	6	13–18	50	Individual semi-structured interview	Interpretative phenomenological analysis
Welsh and Tiffin (135)	At risk for psychosis (Sym)	UK	Child and adolescent mental health clinicians	6	NR	NR	Individual semi-structured interview	Thematic analysis
**Multiple psychological diagnoses**								
Hayne (136)	Mental illness (Sym)	Canada	Individual	14	NR	NR	NR	Hermeneutic phenomenological study; Thematic analysis
McCormack and Thomson (137)	Depression; PTSD (Sym)	Australia	Individual	5	38–62	60	Individual semi-structured interview	Interpretative phenomenological analysis
O'Connor et al. (138)	ADHD, AN, ASD, depression, developmental coordination disorder, non-epileptic seizures (Sym)	Australia, Canada, Denmark, Finland, Hong Kong, Israel, Norway, Puerto Rico, Sweden, UK, USA	Individual	1,083	6–25	NR	Systematic review	Thematic synthesis
Probst (139)	ADHD, AN, Anxiety, ASD, bipolar disorder, depression, dissociative identity disorder, dysthymia, PTSD (Sym)	USA	Individual	30	NR	70	Individual semi-structured interview	Narrative and thematic analysis
Schulze et al. (140)	Schizophrenia (Sym)	Switzerland	Individual	31	23–66	33	Individual interview	Inductive qualitative approach
	BPD (Sym)		Individual	50	18–56	81		
Sun et al. (141)	Psychiatric diagnoses (Sym)	Hong Kong	Psychiatrists	13	NR	15.4	Focus group (*n* = 2)	Conventional content analysis
Perkins et al. (31)	Anxiety, AN BPD, bipolar disorder, depression, schizophrenia, personality disorder, psychosis (Sym)	Australia, Belarus, Brazil, Canada, Denmark, Israel, Latvia, Netherlands, New Zealand, Norway, Sweden, UK, USA	Individual Caregiver Clinicians	NR NR NR	NR NR NR	NR NR NR	Systematic review	Thematic synthesis

* *Conditions organised according to the international classification of diseases 11th edition; Scr, Condition identified through screening; Sym, Condition identified through symptoms; NR, Condition identification methods not reported; Mix, Multiple condition identification methods; GDM, Gestational diabetes mellitus; GERD, Gastro-oesophageal reflux disorder; PCOS, Polycystic ovary syndrome; MRKH, Mayer-rokitansky-kuster-hauser syndrome; HIV, Human immunodeficiency Virus; AIDS, Acquired immunodeficiency syndrome; CFS, Chronic fatigue syndrome; ME, Myalgic encephalitis; MND, Motor neuron disease; HPV, Human papillomavirus; OCD, Obsessive compulsive disorder; AD, Alzheimer's disease; MCI, Mild cognitive impairment; ADHD, Attention deficit hyperactivity disorder; ASD, Autism spectrum disorder; BPD, Borderline personality disorder; PTSD, Posttraumatic stress disorder; AN, Anorexia nervosa; GPs, General practitioners*.

The 44 studies and five reviews includable in our review but not subjected to data extraction due to thematic saturation (final third), had a similar pattern to those used: 28 explored physical and 21 explored psychological diagnostic labels; most reported individual perspectives (76%, 37/49), significantly less reported multiple (12%, 6/49) or family/caregiver perspectives (10%, 5/49), and one (2%) reported healthcare professional or community perspectives. References of these studies are provided in References not subjected to qualitative analyses in Supplementary Material.

### Thematic Synthesis

Qualitative synthesis of included studies identified five overarching themes: psychosocial impact (8 subthemes), support (6 subthemes), future planning, behaviour, and treatment expectations (2 subthemes each). Table 2 reports the number and proportion of records that supported each theme for each of the four perspectives while Table 3 reports the themes and subthemes supported by each included study. Due to the breadth of results, only themes which were supported by >25% of studies, are reported in the text, with themes supported by <25% of articles presented only in tables. Detailed descriptions of all themes and subthemes, with supporting quotes from the individual perspective, are reported in Table 4. Findings from the perspective of family/caregiver, healthcare professionals and community members are briefly reported in text, with details of these themes and supporting quotes reported in Supplementary Tables 1–3, respectively.

**Table 2:** Proportion of records supporting each theme from the various perspectives.

**Major themes**	**Sub themes**	**Description**	**Perspective**			
			**I** **(***n*** = 71)**	**F** **(***n*** = 19)**	**H** **(***n*** = 21)**	**C** **(***n*** = 3)**
Psychosocial impact	Negative psychological impact	Negative psychological impact of labelling	51 (72%)	10 (53%)	7 (33%)	0
	Positive psychological impact	Positive psychological impact of labelling	43 (61%)	5 (26%)	4 (19%)	0
	Mixed psychological impact	Both positive and negative impact of labelling	9 (13%)	3 (16%)	2 (10%)	0
	Psychological adaptation	Psychological adaptation to label and coping strategies/mechanisms	37 (52%)	8 (42%)	1 (5%)	0
	Self-Identity	Changes to self-identity following provision of label (can be positive or negative)	31 (44%)	0	0	0
	Social identity	Changes to social identity as a result of label, including becoming a member/mentor of a support group	28 (39%)	6 (32%)	3 (14%)	2 (67%)
	Social stigma	Perceptions/assumptions of others toward individual labelled	23 (32%)	5 (26%)	2 (10%)	1 (33%)
	Medicalisation	Asymptomatic label and understanding/perception of symptoms	18 (25%)	4 (21%)	6 (29%)	0
Support	Close relationships	Managing relationships and interactions; support required, offered, and accepted following labelling	13 (18%)	8 (42%)	3 (14%)	0
	Healthcare professionals interactions/relationships	Interactions with healthcare professionals; support provided; explanations	32 (45%)	5 (26%)	13 (62%)	0
	Emotional support reduced/limited	Emotional support lost as a result of label or support absent but perceived to be required	26 (37%)	3 (16%)	0	1 (33%)
	Emotional support increased/maintained	Emotional support maintained or increased as a result of label	19 (27%)	5 (26%)	2 (10%)	1 (33%)
	Disclosure	Fear and methods of disclosing label to others (friends/family/employers/colleagues)	26 (37%)	3 (16%)	3 (14%)	0
	Secondary gain	Gains from label	5 (7%)	0	4 (19%)	0
Future planning	Action	Forward planning and decision making as a result of label	12 (17%)	3 (16%)	3 (14%)	0
	Uncertainty	Questions regarding future health and lifestyle	20 (28%)	4 (21%)	0	0
Behaviour	Beneficial behaviour modifications	Behaviour modification/changes as a result of label beneficial to overall health and well-being	21 (30%)	1 (5%)	2 (10%)	0
	Detrimental/unhelpful behaviour modifications	Behaviour modification/changes as a result of label unhelpful/restrictive to overall health and well-being	23 (32%)	9 (47%)	3 (14%)	1 (33%)
Treatment expectations	Positive treatment experiences	Perceptions of treatment/intervention (and outcomes) to be positive/beneficial	20 (28%)	1 (5%)	3 (14%)	0
	Negative treatment experiences	Perceptions of treatment/intervention (and outcomes) to be negative/unhelpful	30 (42%)	5 (26%)	4 (19%)	1 (33%)

*I, Individual perspective; F, Family/Caregiver perspective; H, Healthcare professional perspective; C, Community perspective; Shaded cells represent the numbers of studies that contribute to that theme, Unshaded cells, 0% of studies; Red cells, 1–24% of studies; Yellow cells, 25–49% of studies; Green cells, >50% of studies; one study could reference multiple themes and/or perspectives; Numbers and proportions of studies referenced in the results are calculated from included studies/reviews, with the final third of included studies not included in these tallies*.

**Table 3:** Themes and subthemes supported by each record.

**References (population)**	Condition* (Scr, Sym, NR, Mix)	**Psychosocial impact**								**Support**						**Future planning**		**Behaviour**		**Treatment expectations**	
		**Negative psychological**	**Positive psychological**	**Mixed psychological**	**Psychological adaptation**	**Self-identity**	**Social identity**	**Social stigma**	**Medicalisation**	**Close relationships**	**Healthcare professionals**	**Reduced** **limited**	**Increased maintained**	**Disclosure**	**Secondary gain**	**Action**	**Uncertainty**	**Beneficial modifications**	**Detrimental modifications**	**Positive experiences**	**Negative experiences**
**Cardiovascular disease**																					
Asif et al. (46) (I)	Cardiac conditions (Scr)	✓	✓		✓	✓	✓				✓	✓	✓	✓		✓			✓		✓
**Chronic kidney disease**																					
Daker-White et al. (47) (I)	Chronic kidney disease (Sym)								✓	✓	✓			✓							
**Diabetes**																					
Twohig et al. (48) (I)	Pre-diabetes (Sym)	✓		✓	✓				✓		✓	✓	✓					✓			
Burch et al. (49) (H)	Pre-diabetes (NR)										✓				✓						
de Oliveira et al. (50) (I)	Diabetes (NR)	✓			✓								✓			✓		✓	✓		✓
Due-Christensen et al. (51) (I)	Type 1 diabetes (NR)	✓			✓	✓	✓	✓		✓	✓	✓						✓	✓		
Sato et al. (52) (I)	Type 1 diabetes (NR)	✓			✓	✓						✓		✓			✓				✓
Jackson et al. (53) (F)	Type 1 diabetes (Sym)	✓			✓					✓									✓		
Fharm et al. (54) (H)	Type 2 diabetes (NR)								✓		✓										
Kaptein et al. (55) (I)	GDM (Scr)												✓					✓		✓	✓
Singh et al. (56) (I)	GDM (Scr)	✓					✓					✓						✓			✓
**Female reproduction**																					
Copp et al. (57) (I)	PCOS (Sym)	✓	✓	✓	✓	✓	✓						✓	✓		✓	✓	✓	✓	✓	✓
Copp et al. (58) (H)	PCOS (Sym)	✓	✓	✓				✓	✓		✓					✓		✓	✓	✓	
Newton et al. (59) (I)	Pelvic inflammatory disease (NR)	✓			✓	✓					✓						✓	✓		✓	✓
O'Brien et al. (60) (I)	Anti-Mullerian hormone testing (Scr)	✓	✓			✓					✓					✓	✓				
Patterson et al. (61) (I)	MRKH (Sym)	✓		✓	✓	✓	✓	✓		✓	✓			✓		✓			✓		
Harris et al. (62) (I)	Pre-eclampsia (Scr)	✓			✓				✓								✓	✓	✓	✓	
**Genome/Chromosome**																					
Delaporte (63) (I, H)	Facioscapulohumeral dystrophy (Sym)	✓				✓		✓									✓		✓		
Houdayer et al. (64) (F, H)	Chromosomal abnormalities (Scr)	✓		✓							✓										
**HIV/AIDS**																					
McGrath et al. (65) (I, F)	AIDS (NR)	✓											✓	✓							
Anderson et al. (66) (I)	HIV (NR)	✓	✓				✓					✓					✓		✓		
Freeman (67) (I)	HIV (NR)	✓			✓	✓	✓			✓								✓			
Kako et al. (68) (I)	HIV (NR)	✓			✓		✓						✓	✓							
Kako et al. (69) (I)	HIV (NR)	✓			✓		✓					✓	✓				✓			✓	
Stevens et al. (70) (I)	HIV (NR)	✓			✓														✓		
Firn and Norman (71) (I, H)	HIV/AIDS (NR)				✓			✓				✓									
**Immune system**																					
Hale et al. (72) (I)	Systemic lupus erythematosus (Sym)		✓							✓	✓										✓
**Infectious/Parasitic**																					
Almeida et al. (73) (I)	Leprosy (NR)	✓										✓	✓								
Silveira et al. (74) (I)	Leprosy (NR)	✓			✓			✓				✓	✓	✓							
Zuniga et al. (75) (I)	Tuberculosis (NR)	✓										✓							✓		
Dodor et al. (76) (I, C)	Tuberculosis (NR)					✓	✓	✓				✓									
**Metabolic**																					
Bouwman et al. (77) (I)	Fabry disease (NR)		✓						✓							✓	✓				✓
**Musculoskeletal**																					
Erskine et al. (78) (I)	Psoriatic arthritis (Sym)	✓				✓		✓	✓		✓						✓				
Martindale and Goodacre (79) (I)	Axial spondyloartritis (Sym)	✓	✓	✓	✓																
Hopayian and Notley (80) (I)	Back pain and sciatica (Sym)		✓					✓			✓									✓	✓
Barker et al. (81) (I)	Osteoporosis (Mix)	✓			✓	✓	✓		✓	✓		✓		✓			✓	✓	✓		✓
Hansen et al. (82) (I)	Osteoporosis (NR)	✓		✓	✓									✓				✓	✓		✓
Weston et al. (83) (I)	Osteoporosis (Scr)	✓	✓		✓	✓			✓									✓		✓	✓
Boulton (84) (I)	Fibromyalgia (Sym)		✓						✓		✓										
Madden Sim (85) (I)	Fibromyalgia (Sym)	✓	✓		✓		✓	✓	✓		✓						✓				
Mengshoel et al. (86) (I)	Fibromyalgia (Sym)		✓		✓		✓	✓	✓		✓	✓	✓	✓	✓					✓	✓
Raymond and Brown (87) (I)	Fibromyalgia (Sym)		✓		✓		✓				✓						✓	✓			
Sim Madden y (88) (I)	Fibromyalgia (Sym)	✓	✓		✓	✓	✓	✓		✓	✓	✓		✓			✓	✓	✓	✓	✓
Undeland and Malterud (89) (I)	Fibromyalgia (Sym)		✓					✓			✓	✓		✓	✓						✓
**Nervous system**																					
Chew-Graham et al. (90) and Zarotti et al. (94) (H)	CFS/ME (Sym)	✓	✓	✓					✓				✓								
Hannon et al. (91) (I, F, H)	CFS/ME (Sym)	✓	✓								✓		✓	✓					✓		✓
De Silva et al. (92) (I, F, H, C)	CFS (Sym)		✓					✓													✓
Johnston et al. (93) (I)	MND (Sym)		✓								✓						✓				
Zarotti et al. (94) (H)	MND (Sym)				✓						✓							✓	✓		
Johnson (95) (I)	Multiple sclerosis (Sym)	✓	✓				✓				✓								✓		✓
Thompson et al. (96) (I)	Non-epileptic seizures (Sym)	✓	✓					✓			✓					✓			✓		
Wyatt et al. (97) (I, F)	Non-epileptic attack disorder (Sym)	✓	✓				✓				✓						✓			✓	
**Neurological**																					
Nochi (98) (I)	Traumatic brain injury (Sym)		✓		✓			✓			✓								✓		
Daker-White et al. (99) (I, F)	Progressive ataxias (Sym)	✓	✓				✓		✓	✓					✓	✓					
**Newborn/Foetal**																					
Hallberg et al. (100) (F)	22q11 Deletion syndrome (Scr)	✓	✓	✓			✓			✓			✓			✓	✓		✓		✓
Johnson et al. (101) (F)	Cystic fibrosis (Scr)	✓					✓		✓	✓	✓					✓	✓	✓	✓		
Dahlen et al. (102) (H)	GORD/GERD (Sym)								✓		✓										
**Sleep-Wake disorder**																					
Zarhin (103) (I)	Obstructive sleep apnoea (Sym)	✓				✓			✓												
**Sexually transmitted**																					
Mills et al. (104) (I)	Chlamydia trachomatis (Scr)	✓			✓	✓					✓		✓	✓				✓			
Rodriguez et al. (105) (I)	HPV (NR)	✓	✓	✓	✓	✓	✓	✓		✓	✓	✓	✓	✓			✓	✓	✓	✓	✓
**Multiple physical diagnoses**																					
Kralik et al. (106) (I)	Chronic illness, diabetes (Sym)	✓	✓	✓		✓					✓	✓				✓				✓	
**Bipolar disorder**																					
Fernandes et al. (107) (I)	Bipolar (Sym)	✓	✓		✓	✓	✓	✓		✓	✓	✓	✓	✓				✓	✓	✓	✓
Proudfoot et al. (108) (I)	Bipolar (Sym)	✓	✓		✓	✓		✓				✓		✓			✓				✓
**Depression**																					
Wisdom and Green (109) (I)	Depression (Sym)	✓	✓		✓	✓	✓				✓							✓	✓		✓
Chew-Graham et al. (110) (H)	Depression (Sym)								✓						✓					✓	✓
**Neurocognitive**																					
Beard and Fox (111) (I)	AD; MCI (Sym)		✓		✓	✓	✓		✓	✓						✓					
Bamford et al. (112) (I, F, H)	Dementia (Sym)	✓	✓		✓	✓	✓			✓	✓					✓			✓	✓	
Bunn et al. (113) (I, F)	Dementia (Sym)	✓	✓			✓	✓						✓	✓				✓			✓
Robinson et al. (114) (I, F)	AD; Dementia (Sym)	✓			✓					✓	✓	✓	✓	✓					✓		✓
Ducharme et al. (115) (F)	AD (Sym)	✓	✓		✓			✓						✓			✓		✓		✓
Abe et al. (116) (H)	Dementia (Sym)	✓									✓			✓							
Phillips et al. (117) (H)	Dementia (Sym)	✓								✓	✓			✓	✓	✓					
Walmsley and McCormack (118) (H)	Dementia (Sym)				✓		✓			✓	✓		✓	✓							
Werner and Doron (119) (H, C)	AD (Sym)						✓					✓	✓		✓				✓		
**Neurodevelopmental**																					
Carr-Fanning and Mc Guckin (120) (I, F)	ADHD (Sym)		✓		✓		✓				✓									✓	✓
Mogensen and Mason (121) (I)	ASD (Sym)	✓	✓		✓	✓		✓	✓					✓	✓						
Fleischmann (122) (F)	ASD (Sym)	✓			✓		✓			✓									✓		
Hildalgo et al. (123) (F)	ASD (Sym)							✓			✓										✓
Loukisas and Papoudi (124) (F)	ASD (Sym)	✓			✓			✓	✓	✓		✓				✓	✓		✓		✓
Selman et al. (125) (F)	ASD (Sym)	✓	✓		✓		✓	✓	✓			✓									
Smith et al. (126) (I, F)	ASD (Sym)	✓	✓					✓						✓							
**Obsessive compulsive disorder**																					
Pedley et al. (127) (F)	OCD (Sym)						✓		✓	✓									✓		
**Peri/Postnatal anxiety and/or depression**																					
Ford et al. (128) (H)	Perinatal anxiety and depression (Scr)										✓					✓				✓	✓
Chew-Graham et al. (129) (H)	Postnatal depression (Sym)		✓								✓					✓					✓
**Personality disorder**																					
Horn et al. (130) (I)	BPD (Sym)	✓	✓			✓			✓				✓								✓
Lester et al. (131) (I)	BPD (Sym)	✓	✓						✓		✓										✓
Nehls (132) (I)	BPD (Sym)	✓										✓		✓							✓
**Schizophrenia/Psychotic disorder**																					
Thomas et al. (133) (I)	Schizophrenia (Sym)	✓	✓					✓	✓			✓				✓			✓	✓	
Welsh and Tiffin (134) (I)	At-Risk psychosis (Sym)		✓				✓					✓	✓	✓							
Welsh and Tiffin (135) (H)	At-Risk mental state (Sym)						✓		✓		✓										✓
**Multiple psychological diagnoses**																					
Hayne (136) (I)	Mental illness (Sym)	✓	✓	✓	✓	✓						✓						✓		✓	
McCormack and Thomson (137) (I)	Depression, PTSD (Sym)	✓	✓	✓	✓	✓		✓	✓											✓	
O'Connor et al. (138) (I)	ADHD, AN, ASD, depression, developmental coordination disorder, non-epileptic seizures (Sym)	✓	✓		✓	✓	✓	✓		✓		✓	✓	✓	✓		✓	✓	✓	✓	✓
Probst (139) (I)	ADHD, AN, anxiety, ASD, bipolar disorder, depression, dissociative identity disorder, dysthymia, PTSD (Sym)	✓	✓		✓	✓	✓					✓		✓						✓	
Schulze et al. (140) (I)	Schizophrenia, BPD (Sym)						✓	✓					✓	✓							✓
Sun et al. (141) (H)	Psychiatric diagnoses (Sym)							✓		✓	✓									✓	✓
Perkins et al. (31) (I, F, H)	Anxiety, AN, bipolar disorder, BPD, depression, personality disorder, psychosis, schizophrenia (Sym)	✓	✓	✓		✓	✓	✓		✓	✓		✓				✓			✓	✓
Totals		67	49	14	45	31	38	30	28	24	47	30	26	31	9	19	24	24	34	25	41

I, Individual perspective; F, Family/Caregiver perspective; H, Healthcare professional perspective; C, Community perspective; Cells with “✓” indicate theme explicitly mentioned in the study; Blank cells indicate theme not explicitly mentioned in the study; one study could reference multiple themes and/or perspectives; *Conditions organised according to the International Classification of Diseases 11th edition; Scr, Condition identified through screening; Sym, Condition identified through symptoms; NR, Condition identification methods not reported; Mix, Multiple condition identification methods; GDM, Gestational diabetes mellitus; GERD, Gastro-oesophageal reflux disorder; PCOS, Polycystic ovary syndrome; MRKH, Mayer-Rokitansky-Kuster-Hauser syndrome; HIV, Human immunodeficiency virus; AIDS, Acquired immunodeficiency syndrome; CFS, Chronic fatigue syndrome; ME, Myalgic encephalitis; MND, Motor neuron disease; HPV, Human papillomavirus; OCD, Obsessive compulsive disorder; AD, Alzheimer's disease; MCI, Mild cognitive impairment; ADHD, Attention deficit hyperactivity disorder; ASD, Autism spectrum disorder; BPD, Borderline personality disorder; PTSD, Posttraumatic stress disorder; AN, Anorexia nervosa.

**Table 4:** Major and subthemes arising as consequences for the individual.

**Theme, subtheme, description**	**Exemplary comment**
**Psychosocial impact**	
Negative psychological impact Negative psychological impact of labelling	For some, being seen through the lens of their diagnosis meant being deflated, “robbed of flesh,” crudely translated into an incomplete symbolic language that “*doesn't capture my reality, doesn't see me in my full human complexity, doesn't tell anything substantive about what it's like to actually be me.”* As one person said, “*the diagnosis is like looking at a map of the city but it isn't the city itself” (139)* *That number doesn't sum me up, it doesn't tell the whole storey. I felt offended when I saw it. I didn't feel understood–I felt reduced, diminished. There's nothing in the diagnosis that was really at the heart with what I felt I was afflicted with (139)*
Positive psychological impact Positive psychological impact of labelling	Patients of [Black and Minority Ethnicity] origin described the importance of being believed and taken seriously by their healthcare professionals, and they described how difficult it had been to convince the GPs of their symptoms: “*That is the hardest thing, that is what I find the hardest, even if they didn't find they can cure me, but, just to believe me and have understanding of me, that's all I want” (92)* The diagnosis was used as retaliation against the scepticism encountered within participants' interactions with professionals and the public, and reduced the self-doubt which had been fostered by experiences of being disbelieved. “*Now we've got a label you can turn around and say that's what it is” (97)*
Mixed psychological impact Both positive and negative impact of labelling	Some women shared that they felt relief mixed with fear when a diagnosis was made because they had experienced symptoms that had been very disruptive to their life, and ‘getting diagnosed’ had been a frightening process: *Upon diagnosis I actually felt relief mixed with fear. Relieved because the problem had a name, fearful because there is no cure and no known cause (106)* …she described the conflicting emotions of feeling a sense of relief tempered by the knowledge that this was a long-term condition: ‘*But it’s a double-edged sword, really, because getting the diagnosis is helpful and you know where you stand, and when you talk to people they don't think you are swinging the lead or you are trying to get out of something… but then the flip-side is, oh God, this is me for the rest of my life; it's not going to go away, it's not going to go anywhere' (79)*
Psychological adaptation Psychological adaptation to label and coping strategies/mechanisms	…[diagnosis] eliminated a natural mechanism of coping with stress. This compounded emotional stress related to their diagnosis: “*What I would usually do in a situation like that was run…I was extremely stressed out and because the way I cope with stress is to run and I couldn't run”* (46) Others focused on strategies for symptom management, including “relaxation,” “sleep,” setting “limitations,” “exercise,” and maintaining a “positive attitude” (107)
Self-Identity Changes to self-identity following provision of label (can be positive or negative)	Reconstructing a view of self. This construct referred to how, for many adults in these studies, the diagnosis seemed to change their personal identity which in turn influenced the way they engaged with others and their future aspirations and goals (51) Their perception of themselves had changed so dramatically that, even in a state of physical health after having received curative treatments, they continued to perceive themselves as living with illness (106)
Social identity Changes to social identity as a result of label, including becoming a member/mentor of a support group	Many participants felt that being involved in research allowed them to be proactive, to help advance science, to aid future generations, and to possibly even receive personal benefits (111) Others who had gone public viewed their public acknowledgement of positive [diagnosis]…as a means of reaching others in the community to educate them about [diagnosis] and encourage them to be tested. To these women, disclosure was done out of a sense of duty. They felt they were ambassadors to their communities, even though they risked ridicule and rejection (68)
Social stigma Perceptions/assumptions of others toward individual labelled	They felt disrespected by people who had heard of the diagnosis but still remarked that they did not look ill enough (89) They experienced stigma because of the way the label changed the way other people saw them (133) Besides the image of abnormality, some informants reported that they are considered to be as powerless as children or sick patients (98)
Medicalisation Asymptomatic label and understanding/perception of symptoms	“Normal” vs. “Abnormal” memory loss. Although all respondents acknowledged [symptoms], they had difficulty balancing the “everyday nature of [symptoms]” with the new “reality” that rendered what was previously considered normal, a symptom of disease. Diagnosed individuals were forced to incorporate this tension into their new identities as people living with [symptoms] that was simultaneously the same as past experiences and yet decidedly different (111) The invisible disease. An underlying theme that emerged for many women was the struggle to accept a diagnosis when they felt healthy and had no visible signs of disease. This meant they felt that they had to believe an abstract diagnosis, or they interpreted it as incorrect or insignificant. The absence of visual evidence created mixed reactions to the diagnosis among the women (83)
**Support**	
Close relationships Managing relationships and interactions; support required, offered, and accepted following labelling	Participants also reported a loss of control when their family, friends, or work colleagues engaged in symptom surveillance: *I have actually had friends say, “Are you symptomatic? You are talking a lot. Maybe you have got some [diagnosis]?” (107)* *My boss was really worried that I might have been becoming unwell and, unfortunately, she contacted my psychiatrist before I got there. That was such a breach of confidentiality and just triggered a whole lot of stuff for me.…My boss had said I was wearing different clothes, so it is this fear of, I cannot look different, I cannot wear different things, I cannot have a lot of money or act in certain ways (107)* Loving and caring relationships were felt integral to health and quality of life. Some had become isolated at home or dependent on family and friends for social contact (81)
Healthcare professionals interactions/relationships Interactions with healthcare professionals; support provided; explanations	Some informants felt better understood by health care professionals than by friends or family, whereas others felt misunderstood by the medical profession and society in general. Some informants felt that they were looked upon as being an uninteresting patient, and that once no cure was evident professionals lost patience with them and seemed uninterested and unbelieving (88) They tended to view their health care provider as responsible for “fixing” the problem and did not take responsibility for its remedy. They tended to become frustrated with providers who were not as available as they would like (109)
Emotional support reduced/limited Emotional support lost as a result of labelling; or support absent but perceived to be required	Others were forced out of their communities; they lost some of their friends and family members avoided direct contact with them. (75) Those patients who had experienced a cancelation of their engagement or a divorce because of the disease felt burdened by a handicap that makes them different from others. (52)
Emotional support increased/maintained Emotional support maintained or increased as a result of labelling	Participants thought that their partner, family, friends, health professionals, and support groups provided “advice” and “safety.” For one participant, the support of her husband gave her strength and made her feel “empowered.” Participants also commented on the practical and emotional support they received from friends. For example, one participant stated, “*They used to come and do the washing for me, bring me homemade bread, and look after the family” (107)* Participants consistently described the importance of relationships in terms of hope, recovery and survival. People described how the most significant support they received was from people whom they could trust and who could, as Carol said, “*treat you as a person, rather than a diagnosis” (130)*
Disclosure Fear and methods of disclosing label to others (friends/family/employers/colleagues)	In general, sharing the diagnosis with friends and family was not a problem, though several people expressed anger that they did not have control over the manner, timing, or extent to which this information was shared with employers or other health care providers (139) Other participants discussed the fear they held of losing support people if they told them about their illness. *There are others I would like to share things with, but I don't want to lose anyone else at the present time and it's a risk I'm not willing to take (108)*
Secondary gain Gains from label	Knowing, naming or labelling one's symptoms was also articulated as an important issue in more practical matters such as obtaining benefits or insurance payouts (99) He interpreted this difference positively in terms of the allowances that were sometimes made for him, explaining: ‘*I know that if I wasn’t [diagnosis] my Mum wouldn't let me get away with much stuff'* and ‘*I think I get a bit of easier work’* at school. So although Dylan indicated that the diagnosis was not significant for his self-identity, he recognised that it had a meaning and a function–in perhaps reducing some of the typical school expectations and the way others saw him (121)
**Future planning**	
Action Forward planning and decision making as a result of label	Family planning Some women discussed feeling pressured to have children earlier than they would have liked because they were concerned that if they left it later they would be unable to conceive. A few women did have children earlier than preferred, which was seen to impact on their careers ‘*Yes, that did put the career on hold. I focused on having the children early… I felt with the diagnosis, yeah, you're always thinking about, you know, that fertility side of it. So, yeah, it does affect your decisions’* (57) …felt that an “early” diagnosis made it possible to anticipate future [diagnosis]-related problems, which allowed them to make choices in life “*So you can make conscious decisions: What will I do in life?(…) I am a pharmacist now, so that is not so hard, but what if you have to do something else?(…) If it involves heavy physical activity, you will not be able to do it at a certain point in time. So that is why I feel it is of interest to know” (77)*
Uncertainty Forward planning and decision making as a result of label	…patients indicated that a disadvantage of an early diagnosis was the loss of carefree life and increased worrying about the future. “*Yes, because I have two boys (…) and because I was aware of the medical history in the family, and it's like, well, this is what's in store. My uncle had a couple of kidney transplants and he eventually died of heart failure (…) and then hearing the storeys about my grandmother's brothers–three of them I believe, dying at 35 years of age. Okay, we're talking the turn of the last century of course, but it was disheartening to hear, all the same, and although knowledge of the disease has improved, you still think if you have to go through what my uncle went through, that's not easy”* (77) Fear of what is to come. This describes deep concern with what the future might bring. Hope hinged on success of treatment or being able to successfully accommodate manifestations of [diagnosis] and was countered by fear of unpredictable consequences. Participants described fears of losing mobility, of being wheelchair bound, of being dependent on others and of further fractures, falls and deformity (81)
**Behaviour**	
Beneficial behaviour modifications Behaviour modification/changes as a result of label beneficial to overall health and well-being	Some women acknowledged that developing [diagnosis] was the push they needed to begin adopting healthier behaviour patterns. One woman articulated that diabetes was the “ammunition” her partner needed to encourage her to change her dietary habits and avoid [diagnosis] in the future (55) Although the women did not allow the diagnosis to intrude on their lives, they described themselves as being more sensible than they were previously. These minor adaptations allowed them to manage their increased [symptom] risk but still live as normal. They described taking extra precautions against falling, for example, when it was icy, and they asked for aids such as handrails: *I'm a little more careful in the garden, where I put my tools, where I put my weed bin so I don't fall over it, things like that. We've got quite a large patio with quite a number of steps. I've had a handrail put there and I'm more careful coming down them, whereas I wasn't before…I'm just a little more alert to the dangers if you did fall (83)*
Detrimental/unhelpful behaviour modifications Behaviour modification/changes as a result of label unhelpful/restrictive to overall health and well-being	Another participant thought that she could not be her “usual jolly self” because she feared others would perceive her as being symptomatic of [diagnosis]. Consequently, she thought she had become more “serious” and “less spontaneous,” and she “[thought] twice” about her actions (107) …drug and alcohol use escalated after [diagnosis]. The substance misuse problems they may have had before “really took off” when they found out they had [diagnosis]: *When I went in there and they told me that I was positive, I broke down. I just started drinking and drugging and popping pills. I was devastated. I started severely abusing crack cocaine because it kept the feelings away* (70)
	Along with deep sadness came inactivity, lack of motivation, loss of vigour and initiative, and isolation from family and friends: *I went through depression. I pushed myself away from the family. I had nothing to do with my kids. My sister had to take care of my kids. I was always in my room locked up, crying*. (70)
**Treatment expectations**	
Positive treatment experiences Perceptions of treatment/intervention (and outcomes) to be positive/beneficial	Participants spoke to healing gained from a diagnosis which made illness evident and treatment possible, thus, reinstating them to life (136) Naming experience brought knowledge that there were treatments, which in turn brought hope and a sense of control (139)
Negative treatment experiences Perceptions of treatment/intervention (and outcomes) to be negative/unhelpful	Many participants in our sample were troubled by their medication. Significant concerns were expressed about the negative side-effects and the impact of medication on other areas of their lives, such as blunting their creativity, reducing their energy levels, increasing their weight. Some participants also expressed frustration associated with trialling different medications to find the right combination (108) There was a consistent feeling that diagnosis often led to withdrawal of services, that once this diagnostic decision was made then support was withdrawn (130)

### Individual Perspective

#### Psychosocial Impact

*Psychosocial impact* was identified as the most prevalent theme impacting individuals following being labelled with a diagnostic label. Within this major theme, eight subthemes emerged. *Negative psychological impact, positive psychological impact, and psychological adaptation* were developed with over 50% of studies preferencing the individual's perspective. Subthemes developed with <50% of included articles were *self-identity* (44%)*, social identity* (39%)*, social stigma* (32%)*, medicalisation* (25%), and *mixed psychological impact* (13%) (see Table 2 for overview and Table 4 for details).

#### Negative and Positive Psychological Impact

Both positive and negative consequences of diagnostic labelling to individuals were reported. Almost 72% of studies describing consequences of labelling from the individual's perspective reported negative psychological consequences including resistance, shock, anxiety, confusion, bereavement, abandonment, fear, sadness, and anger frequently reported (46, 50–52, 56, 57, 59–63, 65, 66, 68–70, 74, 75, 81, 82, 85, 88, 92, 95–97, 99, 103–106, 108, 112, 113, 126, 136, 138, 139). Conversely, 61% of studies reported a positive psychological impact of being provided with a diagnostic label. For example, many individuals reported that receiving a diagnostic label produced feelings of relief, validation, legitimisation, and empowerment (31, 46, 57, 60, 66, 72, 77, 79, 80, 83, 84, 86–89, 91, 92, 96, 97, 99, 105–109, 111, 113, 120, 121, 126, 133, 134, 136, 139). Other studies reported individuals described diagnostic labels as providing hope and removing uncertainty (93, 95, 96, 112, 121, 130, 134, 136, 137), facilitating communication with others (98, 130), and increasing self-understanding (97, 131, 138).

#### Psychological Adaptation

Upon receipt of a diagnostic label, 52% of included studies from an individual's perspective reported a need to change their cognitions and emotions. Included studies reported individuals described adaptive (e.g., using humour) and maladaptive (e.g., suicidality) coping mechanisms (46, 48, 50, 57, 61, 67–69, 71, 74, 82, 85, 88, 98, 105, 107–109, 111, 112, 114, 136, 138, 139), adapting to new condition-specific knowledge (62, 79, 87, 88, 121), rejecting negative perceptions (50, 51, 70, 104, 138), and accentuating positive elements of the condition (51, 52, 61, 86, 105, 111). These adaptations were reported to be centred around living fulfilling lives post diagnostic labelling (70, 83, 88, 107).

Changes to *self-identity* was reported by individuals in 44% of included studies. These studies reported individuals experienced a disruption to their perception of self and previously held identities (46, 51, 57, 59, 61, 78, 81, 103, 104, 107, 113, 136, 137, 139). Some of these changes were viewed constructively, including reported perceptions of empowerment, transformation, and self-reinforcement (51, 67, 83, 88, 107, 109, 121, 137–139). Others, however, reported negative impacts such as enforced separation from those who did not have a label, and perceptions of themselves as unwell and less competent (31, 51, 52, 60, 63, 76, 88, 105–107, 109, 111–113, 121, 136, 138, 139).

Changes to *social identity* and experiences of *social stigma* were reported in 39% and 32% of included studies, respectively. Within newly developed social identities, mentorship and support groups were frequently reported as beneficial (31, 46, 51, 56, 57, 68, 69, 81, 85–88, 97, 107, 109, 111, 113, 134, 138, 139), although sometimes not (61, 85, 107, 113). In some studies, individuals perceived increased stigmatisation, including judgement, bullying, powerlessness, isolation, and discrimination, from families, friends, and society (31, 51, 61, 63, 74, 78, 85, 98, 105, 107, 108, 121, 133, 137, 138), and healthcare professionals (88, 133). Few studies reported individuals perceived their diagnostic label negatively impacted employment (71, 76, 138).

A quarter of the studies reporting individual perspectives, referenced the concept of *medicalisation* at various points along the diagnostic labelling pathway. For example, at the point of diagnostic labelling, some individuals described the diagnostic label as medicalising their asymptomatic diagnosis (71, 76, 138), others struggled with differentiating normal and abnormal experiences (99, 111), while others attributed all symptoms and behaviours to the provided diagnostic label (85, 86, 121, 133).

#### Support

Within this major theme, six subthemes emerged. The most frequently reported was individuals' *interactions with healthcare professionals* in 45% of included studies. Fewer studies reported on *disclosure* (37%), or changes in the perceived or actual support received following receipt of a diagnostic label with *loss of support* reported in 37% of studies and *increased support* reported in 27% of studies. *Close relationships* and *secondary gains* were less prevalent themes reported in <25% of included studies.

*Healthcare professional interactions* were reported to occur along a spectrum from individuals feeling adequately supported and reassured (31, 46, 51, 59, 60, 87, 93, 95, 96, 131) through to individuals feeling dismissed and not listened to (31, 59, 61, 72, 78, 80, 84–86, 89, 91, 93, 95, 97, 98, 104–107, 120). Perception of interactions with healthcare professionals often reflected the individual's understanding of the healthcare professionals': role [e.g., responsible for correcting the diagnosis, open discussion between professional and individual (47, 109)]; the perceived level of skill, knowledge and competency (95, 97); and communication skills (47, 91, 112).

Individuals *disclosing* their diagnostic label to others was a dilemma reported in 37% of included studies. Concerns about whether, when and to whom to disclose where frequently reported (46, 47, 57, 61, 104, 105, 132, 134, 139, 140). Reasons for hesitation included worry, shame, and embarrassment (65, 81), fear of rejection or loss of support (52, 61, 65, 68, 74, 105, 108), anticipation of stigma (65, 68, 86, 88, 89, 105, 121); loss of pre-diagnostic labelled self (82, 107, 113, 138), and fear of losing employment (74, 86, 138). Disclosure was often reported to occur out of a “sense of obligation” (68, 91, 126, 134, 138).

As a result of the diagnostic label, individuals in the included studies reported *similar, increased*, and *decreased emotional support*. Some individuals reported others became more emotionally and physically distant, either overtly or covertly, and more stigmatising (48, 51, 56, 69, 71, 73–76, 81, 88, 89, 105, 107, 108, 133, 134, 136, 138) following label disclosure, some experienced breakdowns of romantic relationships and marriages (52, 66, 105, 107), and some perceived a reduction in support from healthcare professionals following diagnostic labelling (46, 56, 86, 106, 132, 133, 136, 139). In contrast, others indicated no change or an increase in support from family, friends, and communities, reporting acceptance, tolerance, and strengthened relationships (31, 46, 48, 50, 55, 57, 68, 69, 73, 74, 86, 91, 105, 107, 113, 130, 134, 138, 140).

#### Future Planning

Within this major theme, two subthemes emerged which were related to the certainty of future aspirations and planning: *uncertainty* (28%) and *action* (imminent need or ability to respond, 17%).

Individuals who reported *uncertainty* about their future health and lifestyles reported fear, worry, stress, anxiety, and passivity around their futures (57, 69, 88, 97), with these emotions related to changes to life-plans (66, 69, 77, 108, 138), including reproductive abilities (57, 59, 60, 105), potential complications due to the diagnostic label and/or its treatment (52, 57, 62, 63, 69, 81), and unclear disease progressions (31, 77, 78, 85, 87, 93).

#### Behaviour Modification

*Behaviour modification* was reported as either *beneficial* to greater overall health and well-being (reported in 30% of included studies) or *detrimental* and perpetuated or exacerbated condition difficulties (reported in 32%).

*Beneficial behaviour modifications* included greater ownership of health (51, 82, 109, 136) and positive changes to physical activity practises, dietary choices, self-awareness, and risk management (48, 50, 51, 55–57, 59, 62, 67, 81–83, 87, 88, 104, 105, 107, 109, 113, 136, 138). While *detrimental behaviour modifications* were reported as activity restriction (46, 51, 66, 88, 105, 107, 112, 133), reduction in employment and educational opportunities (63, 81, 107, 133, 138), and withdrawal from social interactions and relationships (51, 61, 66, 74, 75, 81, 95, 96, 105). Other individuals indicated increased hypervigilance (51, 57, 75, 112) and additional disruptive and risk-taking behaviours (50, 57, 70, 82, 98) and suicide attempts (70, 107, 138).

Following receipt of a diagnostic label, ***treatment expectations*** were reported by some individuals as both *positive* (reported in 28% of included studies) and *negative treatment experiences* (42%). Some individuals reported condition labelling facilitated access to treatment, monitoring, and support (31, 55, 57, 59, 62, 69, 86, 106, 112, 133, 136–138), which produced hope, empowerment, and perceived control (31, 80, 83, 88, 97, 105, 139) and contributed to *positive treatment experiences*. Contributing to *negative treatment* experiences, however, others indicated the labels failed to guide treatment (31, 57, 59, 77, 80, 86, 89, 95, 105, 114, 132), and that treatments were ineffective, difficult to sustain, and had detrimental effects (46, 50, 52, 55, 56, 77, 80–83, 88, 91, 105, 107–109, 113, 120, 131, 138); and lack of control over (72, 107, 140), or rejection from services (31, 95, 130–132).

### Perspectives of Family/Caregivers, Healthcare Professionals, and Community Members

Fewer studies reported consequences of a diagnostic label from the perspectives of **family/caregivers** (*n* = 19 studies), **healthcare professionals** (*n* = 21 studies) and **community perspectives** (*n* = 3 studies; Table 2 for overview and Supplementary Tables 1–3, respectively, for details). **Family/caregivers** primarily reported *negative psychological impacts* of diagnostic labelling (53%). Other subthemes comprised evidence from <50% of included articles, including *detrimental behaviour modifications* (47%), *psychological adaptation* and *close relationships* (42%), *social identity* (32%), and *positive psychological impact, social stigma, healthcare professional interactions/relationships, increase/maintained emotional support*, and *negative treatment experiences* (all 26%).

**Healthcare professionals** predominantly reported on their *interactions/relationships* (62%) with patients following diagnostic labelling, the potential *negative psychological impact* (33%) a diagnostic label would have and how this could lead to *medicalisation* (29%) of symptoms.

Although the **community** perspective was least frequently reported, two-thirds of the included studies (67%) reported the diagnostic label had an impact on the *social identity* of the individual labelled. Single studies from the community perspective reported themes of *social identity, social stigma, increased/maintained emotional support, reduced/limited emotional support, detrimental/unhelpful behaviour modifications*, and *negative treatment experiences* (all 33%). No studies from the community perspective supported the remaining 14 subthemes.

## Discussion

The findings from our systematic scoping review identified a diverse range of consequences of being labelled with a diagnostic label that vary depending on the perspective. Five primary themes emerged: *psychosocial impact, support, future planning, behaviour, and treatment expectations*, with each theme having multiple subthemes. All five primary themes were reported from each perspective: individual; family/caregiver; healthcare professional; or community member. Within each primary theme there were examples of both positive and negative impacts of the diagnostic label.

However, the developed framework suggests that receiving a diagnostic label is not solely beneficial. For example, of the studies in our review which reported a psychosocial consequence of a diagnostic label, 60% of these reported negative psychological impacts, compared with 46% that reported positive psychological impacts. The results of this review also suggest many individuals experience changes in their relationships with healthcare providers (and the latter agreed), lost emotional support, and experienced a mix of both beneficial and detrimental changes in behaviour due to the diagnostic label.

### Strengths and Limitations

A strength of the current review is the inclusivity of consumers in the development of the initial framework through social media polling, which increased the breadth of the search strategy, and embedded consumers perspective into the developed framework. Inclusion of both physical and psychological diagnostic labels and data from multiple perspectives (i.e., individual, family/caregiver, healthcare professional, community members) addresses limitations of previous studies and increases the generalisability of the findings (30–32). Further, examining varied perspectives highlighted the diverse impact of diagnostic labelling and both common and lesser reported or explored consequences. The staged process of data extraction provided an opportunity to refine and validate the framework, with separate reporting of qualitative and quantitative results allowing for a more thorough discussion of findings. The random process used to extract data resulted in studies selected for extraction having similar characteristics (e.g., physical, psychological, proportion reporting on each perspective) to those articles which were not selected (i.e., last third). Therefore, the articles synthesised in the framework are representative of all articles included in the review.

There are several limitations which might impact the interpretations of our results. First, the volume of retrieved and included studies in this review resulted in pragmatic decisions regarding the separation of reporting qualitative and quantitative findings. As this is a scoping review, the methodological quality of included studies was not assessed which may impact the interpretation of these results. Although our scoping review did not include grey literature and non-peer reviewed research (e.g., dissertations), we believe the volume of included studies and achievement of data saturation for the thematic coding make novel findings from these sources unlikely. While our findings can be generalised to a large number physical and psychological diagnoses, they cannot be extended to cancer diagnoses. The decision to exclude cancer diagnoses was due to an existing body of literature that documents consequences of cancer diagnoses, the increased perceived severity and lethality of cancer diagnoses, and assumptions of increased invasiveness of treatments (37–39). Considering the expanse of research available in the field of cancer, and the potential for this literature to dominate the articles included and synthesised in this review, cancer diagnoses were excluded (37–39). Lastly, time since diagnostic labelling could not be determined in many of the studies included in this review. Time since diagnostic labelling may have various impacts on diagnostic label consequences, with the potential for consequences to increase, and/or decrease, in severity over time.

Individual perspectives of the consequences of diagnostic labelling have been more thoroughly researched than the perspectives of family/caregivers, healthcare professionals or community members. Although one could argue this is reasonable, the paucity of research exploring healthcare professional perspectives is surprising given these individuals are currently primarily responsible for the provision of diagnostic labels. Failure to thoroughly examine consequences of diagnostic labelling from these perspectives may serve to perpetuate harms, including stigma and overtreatment, for certain diagnoses. Exploring the consequences from these lesser represented perspectives would be a valuable area for future research.

### Study Results in Relation to Other Reviews

The findings of our review confirm and expand those of other reviews, including highlighting the range of psychological impacts of receiving a diagnostic label (e.g., positive, negative, mixed), changes to self-identity of the individual labelled, and the questioning of condition prognosis (15, 142). While the current review excluded cancer conditions, the results of our review confirm those of Nickel et al. (39) who found that, in hypothetical case scenarios of medicalized, compared to descriptive, terminology for both cancer and non-cancer diagnoses, the provision of a diagnostic label may have detrimental psychological impacts, including increased anxiety, increased perceived severity of the diagnosis, and preference for more invasive treatments. Further, existing reviews investigating the impact of cancer diagnosis on individuals and family members (143, 144) support findings of the current review, including the varied psychological impacts and impacts on support and treatment decisions. Our review also extends these findings first, across multiple diagnostic labels (e.g., diabetes, musculoskeletal, and autism spectrum disorder) and second, using real-world experiences (39). Our review also confirms the precedents proposed by social constructionism, labelling, and modified labelling theories, which suggest diagnostic labelling activates multifaceted responses, including impacting multiple areas of an individuals' well-being and identity as well as evoking a range of societal assumptions (3, 20–22).

### Clinical Implications

Overall, there is a need for individuals, family/caregivers, healthcare professionals and community members to be more aware of the potential consequences of diagnostic labels in addition to increased discussion of these impacts at the point of, or prior to, provision of diagnostic labels. While normative practise may overlook the impact receiving a diagnostic label, increasing awareness of the potential consequences, both positive and negative, may increase judicious use of diagnostic labels to ensure greatest benefit and least harm, for individuals, families and caregivers, and wider health systems. In the context of overdiagnosis and expanding disease definitions, such discussion, and decided use of, diagnostic labels is particularly pertinent for individuals being diagnosed with mild symptoms or characteristics indicative of asymptomatic diagnostic labels.

With further evaluation, it is anticipated that our framework could form the basis for discussions prior to the provision of a diagnostic label to increase individuals' awareness of the potential psychosocial, behavioural and relationship changes, expectations about treatments, and future planning associated with the diagnostic label. Elements of the framework, in conjunction with the Checklist to Guide Modification of Disease Definitions, developed by Doust et al. (145), may also be used by panels to consider the impacts of a diagnostic label before modifying existing diagnostic criteria, particularly when planning to lower thresholds for diagnosis. Further, researchers' consideration of the developed framework may allow for increasingly targeted research objectives, inclusive of wide-ranging possible impacts, which serve to inform modifications to diagnostic criteria, treatment guidelines, and healthcare professional training programs. Considering the diverse consequences associated with a diagnostic label, a discussion to review how healthcare services and support are allocated, for example, channelling resources away from condition-specific allocation and toward a needs-based allocation, is worthwhile.

Additionally, there is a role for shared decision making (SDM) at the point of diagnostic labelling for individuals who are asymptomatic or present with mild symptoms. In such instances, information about the consequences of receiving a diagnostic label could be provided to the individual and their family/caregiver as a discussion aid, a tool that can facilitate SDM, prior to the provision of a diagnostic label. This information would potentially enable a discussion to ensue about whether (or not) diagnostic label is necessary and beneficial given the individual's circumstances (146, 147). Such a discussion between the individual and healthcare professional may effectively circumvent an individual receiving a diagnostic label, or prepare an individual for the potential psychosocial, relational, behavioural, and treatment consequences following receipt of a diagnostic label.

### Future Research

The developed framework proposes a range of potential consequences of diagnostic labelling. However, additional research is required to continue to validate and develop the framework, particularly from healthcare professional and community perspectives. It would be interesting to examine these less explored perspectives as further insights into the experience of diagnostic labelling may provide additional aspects to the developed framework.

Further research is required to determine the impact of health symptom severity and prognosis on receiving a diagnostic label. Synthesis of research exploring the consequences of receiving a cancer diagnosis (not addressed in this review) will determine the applicability of the framework to cancer conditions and examine the similarities and differences between labelling cancer and non-cancer condition, potentially adding to the current framework. As we excluded studies that explored the consequences of a cancer diagnosis (often thought to be life-threatening diagnoses), we do not know whether consequences of “life-threatening” diagnostic labelling differ from other diagnostic labels. Exploration of these areas may be beneficial in further developing the framework and considering its generalisability.

The framework developed in our systematic scoping review synthesises the consequences of a diagnostic label that are applicable to both physical and psychological diagnostic labels. The findings of this review promote the need for individuals, family/caregivers, healthcare professionals, and community members to be more aware of, and openly discuss, the consequences of a diagnostic label before a diagnosis is made. In a time when diagnostic labels are often rapidly and frequently provided, and healthcare resources are increasingly scarce, there is a growing need to promote the judicious use of diagnostic labels for those who are most likely to benefit.

## Author Contributions

RS, PG, and RT contributed to the conception and design of the study, initial public polling survey on social media and search term construction. RS and ZAM contributed to screening and data analysis. RS, ZAM, RT, and PG contributed to the drafting of the manuscript. All authors approved the final version.

## Funding

RS was supported by an Australian Government Research Training Program Scholarship. RT and ZAM are supported by a National Health and Medical Research Council Program grant (#1106452). PG was supported by a NHMRC Research Fellowship (#1080042). The funding sources have no role in study design, data collection, data analysis, data interpretation, or writing of the report.

## Conflict of Interest

The authors declare that the research was conducted in the absence of any commercial or financial relationships that could be construed as a potential conflict of interest.

## Publisher's Note

All claims expressed in this article are solely those of the authors and do not necessarily represent those of their affiliated organizations, or those of the publisher, the editors and the reviewers. Any product that may be evaluated in this article, or claim that may be made by its manufacturer, is not guaranteed or endorsed by the publisher.
