# Supplementary material for: Consequences of a Diagnostic Label: A Systematic Scoping Review and Thematic Framework
Source: Front Public Health. 2021 Dec 22;9:725877. doi: 10.3389/fpubh.2021.725877 (PMC8727520; doi:10.3389/fpubh.2021.725877)
Supplement: Supplementary file 1 [file Data_Sheet_1.PDF]

Supplementary Material

1 Supplementary Data

1.1 PubMed Search Strategy

(Health[tiab] OR Illness[tiab] OR Disorder[tiab] OR Condition[tiab] OR Disease[tiab])

AND

((Psychological[ti] OR Label[tiab] OR Labelling[tiab] OR Labeling[tiab]) AND (Diagnosis[tiab] OR Diagnostic[tiab] OR Screening[Mesh] OR Screening[tiab] OR Screened[tiab]))

AND

(Patient[tiab] OR Patients[tiab] OR Individuals[tiab] OR Self[tiab] OR Parent[tiab] OR Family[tiab] OR Adult[tiab] OR Men[tiab] OR Women[tiab])

AND

(Attitude[Mesh] OR Awareness[tiab] OR Stigma[tiab] OR Beliefs[tiab] OR Well-being[tiab] OR Wellbeing[tiab] OR Meaning[tiab] OR Impact[tiab] OR Effect[tiab] OR Effects[tiab] OR Influence[tiab] OR Experience[tiab])

AND

("Systematic review"[tiab] OR "Systematic Review"[pt] OR "Cochrane Database Syst Rev"[ta] OR "meta analysis"[pt] OR "meta analysis"[tiab] OR ((Search[tiab] OR Searched[tiab] OR Searches[tiab]) AND (PubMed[tiab] OR Medline[tiab] OR Database[tiab] OR Databases[tiab])) OR "randomized controlled trial"[pt] OR "controlled clinical trial"[pt] OR randomized[tiab] OR randomised[tiab] OR placebo[tiab] OR randomly[tiab] OR trial[tiab] OR groups[tiab] OR "Epidemiologic Studies"[Mesh] OR "case-control studies"[Mesh] OR "Cohort Studies"[Mesh] OR "case control"[tiab] OR Cohort[tiab] OR "Follow up"[tiab] OR Observational[tiab] OR Longitudinal[tiab] OR Prospective[tiab] OR retrospective[tiab] OR "cross sectional"[tiab] OR "Cross-Sectional Studies"[Mesh] OR Investigated[tiab] OR Analysis[tiab] OR Statistics[tiab] OR Data[tiab] OR "statistics and numerical data"[sh] OR "epidemiology"[sh])

NOT

(Animals[Mesh] NOT (Animals[Mesh] AND Humans[Mesh]))

NOT

(Injections[Mesh] OR Open-Label[tiab] OR "Product Labeling"[Mesh] OR "Drug Labeling"[Mesh] OR "Affinity Labels"[Mesh] OR "Food Labeling"[Mesh] OR "Isotope Labeling"[Mesh] OR "Staining and Labeling"[Mesh] OR "In Situ Nick-End Labeling"[Mesh] OR "Primed In Situ Labeling"[Mesh] OR Rat[ti] OR Rats[ti] OR Mice[ti] OR Mouse[ti] OR Placebo[ti] OR "Drug effects"[sh] OR Drug[ti] OR Drugs[ti] OR "Food and Drug Administration"[ti] OR "Food labeling"[ti] OR "Calorie labeling"[ti] OR Injection[ti] OR Cigarette[ti])

1.2 References not subjected to qualitative analyses

1. Adriaanse MC, Snoek FJ, Dekker JM, van der Ploeg HM, Heine RJ. Screening for Type 2 diabetes: an exploration of subjects' perceptions regarding diagnosis and procedure. *Diabet Med* 2002;19:406-11. doi:10.1046/j.1464-5491.2002.00710.x.
2. Aoun SM, O'Brien MR, Breen LJ, O'Connor M. 'The shock of diagnosis': qualitative accounts from people with motor neurone disease reflecting the need for more person-centred care. *J Neurol Sci* 2018;387:80-84. doi:10.1016/j.jns.2018.01.026.
3. Calzada LR, Pistrang N, Mandy WPL. High-functioning autism and asperger's sisorder: utility and meaning for families. *J Autism Dev Disord* 2012;42:230-43. doi:10.1007/s10803-011-1238-5.
4. Champlin BE. The informal caregiver's lived experience of being present with a patient who receives a diagnosis of dementia: a phenomenological inquiry. *Dementia* 2020;19:375-96. doi:10.1177/1471301218776780.
5. Cooper S, Gilbert L. An exploratory study of the experience of fibromyalgia diagnosis in South Africa. *Health* 2017;21:337-53. doi:10.1177/1363459316677623.
6. Cotter AR, Vuong K, Mustelin LL, et al. Do psychological harms result from being labelled with an unexpected diagnosis of abdominal aortic aneurysm or prostate cancer through screening? A systematic review. *BMJ Open* 2017;7:e017565. doi:10.1136/bmjopen-2017-017565.
7. Culley L, Law C, Hudson N, et al. The social and psychological impact of endometriosis on women's lives: a critical narrative review. *Hum Reprod* 2013;19:625-39. doi:10.1093/humupd/dmt027.
8. Daley EM, Perrin KM, McDermott RJ, et al. The psychosocial burden of HPV: a mixed-method study of knowledge, attitudes and behaviors among HPV plus women. *J Health Psychol* 2010;15:279-90. doi:10.1177/1359105309351249.
9. Darroch J, Myers L, Cassell J. Sex differences in the experience of testing positive for genital chlamydia infection: a qualitative study with implications for public health and for a national screening programme. *Sex Transm Infect* 2003;79:372-74. doi:10.1136/sti.79.5.372.
10. Due-Christensen M, Willaing I, Ismail K, Forbes A. Learning about type 1 diabetes and learning to live with it when diagnosed in adulthood: two distinct but inter-related psychological processes of adaptation. A qualitative longitudinal study. *Diabet Med* 2019;36:742-52. doi:10.1111/dme.13838.
11. Dures E, Bowen C, Brooke M, et al. Diagnosis and initial management in psoriatic arthritis: a qualitative study with patients. *Rheumatol Adv Pract* 2019;3:rkz022. doi:10.1093/rap/rkz022.
12. Edwards E, Timmons S. A qualitative study of stigma among women suffering postnatal illness. *J Ment Health* 2009;14:471-81. doi:10.1080/09638230500271097.
13. Elkington KS, Hackler D, McKinnon K, Borges C, Wright ER, Wainberg ML. Perceived mental illness stigma among youth in psychiatric outpatient treatment. *J Adolesc Res* 2012;27:290-317. doi:10.1177/0743558411409931.
14. Finnegan R, Trimble T, Egan J. Irish parents' lived experience of learning about and adapting to their child's autistic spectrum disorder diagnosis and their process of telling their child about their diagnosis. *Ir J Psychol* 2014;35:78-90. doi:10.1080/03033910.2014.982143.
15. Floris J, McPherson S. Fighting the whole system: dissociative identity disorder, labeling theory, and iatrogenic doubting. *J Trauma Dissociation* 2015;16:476-93. doi:10.1080/15299732.2014.990075.

16. Gambling T, Long AF. An exploratory study of young women adjusting to developmental dysplasia of the hip and deciding on treatment choices. *Chronic Ill* 2012;8:17-30. doi:10.1177/1742395311417638.
17. Giovannetti AM, Brambilla L, Torri Clerici V, et al. Difficulties in adjustment to multiple sclerosis: vulnerability and unpredictability of illness in the foreground. *Disabil Rehabil* 2017;39:897-903. doi:10.3109/09638288.2016.1170212.
18. Hagan RJ. What next? Experiences of social support and signposting after a diagnosis of dementia. *Health Soc Care Community* 2020;28:1170-79. doi:10.1111/hsc.12949.
19. Han S, Middleton PF, Bubner TK, Crowther CA. Women's views on their diagnosis and management for borderline gestational diabetes mellitus. *J Diabetes Res* 2015;2015:209215. doi:10.1155/2015/209215.
20. Harris JM, Franck L, Michie S. Assessing the psychological effects of prenatal screening tests for maternal and foetal conditions: a systematic review. *J Reprod Infant Psychol* 2012;30:222-46. doi:10.1080/02646838.2012.710834.
21. Hendriks KS, Grosfeld FJ, van Tintelen JP, et al. Can parents adjust to the idea that their child is at risk for a sudden death? Psychological impact of risk for long QT syndrome. *Am J Med Genet* 2005;138a:107-12. doi:10.1002/ajmg.a.30861.
22. Hickey A, Crabtree J, Stott J. 'Suddenly the first fifty years of my life made sense': experiences of older people with autism. *Autism* 2018;22:357-67. doi:10.1177/1362361316680914.
23. Holt RE, Slade P. Living with an incomplete vagina and womb: an interpretative phenomenological analysis of the experience of vaginal agenesis. *Psychol, Health Med* 2003;8:19-33. doi:10.1080/1354850021000059232.
24. Hugel H, Grundy N, Rigby S, Young CA. How does current care practice influence the experience of a new diagnosis of motor neuron disease? A qualitative study of current guidelines-based practice. *Amyotroph Lateral Scler* 2006;7:161-66. doi:10.1080/14660820600601051.
25. Huggett C, Birtel MD, Awenat YF, et al. A qualitative study: experiences of stigma by people with mental health problems. *Psychol Psychother* 2018;91:380-97. doi:10.1111/papt.12167.
26. Huws JC, Jones RSP. Diagnosis, disclosure, and having autism: an interpretative phenomenological analysis of the perceptions of young people with autism. *J Intellect Dev Disabil* 2008;33:99-107. doi:10.1080/13668250802010394.
27. Jacob JD, Gagnon M, McCabe J. From distress to illness: a critical analysis of medicalization and its effects in clinical practice. *J Psychiatr Ment Health Nurs* 2014;21:257-63. doi:10.1111/jpm.12078.
28. Kahn JA, Slap GB, Bernstein DI, et al. Psychological, behavioral, and interpersonal impact of human papillomavirus and pap test results. *J Womens Health* 2005;14:650-9. doi:10.1089/jwh.2005.14.650.
29. Klasen H. A name, what's in a name? The medicalization of hyperactivity, revisited. *Harv Rev Psychiatry* 2000;7:334-44. doi:10.3109/hrp.7.6.334.
30. Ladd W. “Born out of fear”: a grounded theory study of the stigma of bipolar disorder for new mothers. *Qual Rep* 2018;23:2081-104. doi:10.46743/2160-3715/2018.3382.
31. Lempp HK, Hatch SL, Carville SF, Choy EH. Patients' experiences of living with and receiving treatment for fibromyalgia syndrome: a qualitative study. *BMC Musculoskelet Disord* 2009;10:124. doi:10.1186/1471-2474-10-124
32. Lewis LF. Realizing a diagnosis of autism spectrum disorder as an adult. *Int J Ment Health Nurs* 2016;25:346-54. doi:10.1111/inm.12200.

33. Low L-F, Swaffer K, Brodaty H, Brodaty H. Communicating a diagnosis of dementia: a systematic mixed studies review of attitudes and practices of health practitioners. *Dementia* 2019;18:2856-905. doi:10.1177/1471301218761911.
34. Midence K, O'Neill M. The experience of parents in the diagnosis of autism. A pilot study. *Autism* 1999;3:273-85. doi:10.1177/1362361399003003005.
35. O'Brien MR, Whitehead B, Jack BA, Mitchell JD. From symptom onset to a diagnosis of amyotrophic lateral sclerosis/motor neuron disease (ALS/MND): experiences of people with ALS/MND and family carers - a qualitative study. *Amyotroph Lateral Scler* 2011;12:97-104. doi:10.3109/17482968.2010.546414.
36. Pesonen H-M, Remes AM, Isola A. Diagnosis of dementia as a turning point among Finnish families: a qualitative study. *Nurs Health Sci* 2013;15:489-96. doi: 10.1111/nhs.12059.
37. Portway SM, Johnson B. Do you know I have asperger's syndrome? Risks of a non-obvious disability. *Health Risk Soc* 2005;7:73-83. doi:10.1080/09500830500042086.
38. Rafael F, Houinato D, Nubukpo P, et al. Sociocultural and psychological features of perceived stigma reported by people with epilepsy in Benin. *Epilepsia* 2010;51:1061-8. doi:10.1111/j.1528-1167.2009.02511.x.
39. Rose D, Thornicroft G. Service user perspectives on the impact of a mental illness diagnosis. *Epidemiol Psychiatr Sci* 2010;19:140-7. doi:10.1017/s1121189x00000841.
40. Russell L, Moss D. High and happy? Exploring the experience of positive states of mind in people who have been given a diagnosis of bipolar disorder. *Psychol Psychother Theory Res Pract* 2013;86(4):431-46. doi:10.1111/j.2044-8341.2012.02064.x.
41. Sanderson SC, Linderman MD, Suckiel SA, et al. Psychological and behavioural impact of returning personal results from whole-genome sequencing: the HealthSeq project. *Eur J Hum Genet* 2017;25:280-92. doi:10.1038/ejhg.2016.178.
42. Smyth KM, Salloum AA. Secrecy, adaptation, and liminality in early-onset bipolar disorder: reflections from a sample of emerging adults. *Soc Work Ment Health* 2019;17:723-42. doi:10.1080/15332985.2019.1666079.
43. Tewksbury R, McGaughey D. Identities and identity transformations among persons with HIV disease. *J Gay Lesbian Bisexual Identity* 1998;3:213-32. doi:10.1023/A:1023243032307.
44. Travell C, Visser J. 'ADHD does bad stuff to you': young people's and parents' experiences and perceptions of attention deficit hyperactivity disorder (ADHD). *Emot Behav Diffic* 2006;11:205-16. doi:10.1080/13632750600833924.
45. Troughton J, Jarvis J, Skinner C, Robertson N, Khunti K, Davies M. Waiting for diabetes: perceptions of people with pre-diabetes. A qualitative study. *Patient Educ Couns* 2008;72:88-93. doi:10.1016/j.pec.2008.01.026.
46. Twist K, Ablett J, Wearden A, et al. Gastrointestinal dysmotility: a qualitative exploration of the journey from symptom onset to diagnosis. *J Neurogastroenterology Motil* 2018;30:e13339. doi:10.1111/nmo.13339.
47. Waldron N, Brown SJ, Hewlett S, Elliott, B, McHugh N, McCabe CS. 'To suddenly have a name for this thing... was wonderful': the patient's experience of receiving a diagnosis of systemic lupus erythematosus. *Musculoskelet Care* 2012;10:135-41. doi:10.1002/msc.1010.
48. Whittemore R, Jaser S, Chao A, Jang M, Grey M. Psychological experience of parents of children with type 1 diabetes: a systematic mixed-studies review. *Diabetes Educ* 2012;38:562-79. doi:10.1177/0145721712445216.

49. Woodward RV, Broom DH, Legge DG. Diagnosis in chronic illness: disabiling or enabling - the case of chronic fatigue syndrome. *J R Soc Med* 1995;88:325-29. doi:10.1177/014107689508800606.

2 Supplementary Tables

2.1 Supplementary Table 1 Major and subthemes arising as consequences for the family/caregiver

**Theme,** *Subtheme,* Description	**Exemplary comment**
**Psychosocial impact**	
Negative psychological impact<br>Negative psychological impact of labelling	All parents also described the sorrow they felt when they got the diagnosis, because it was final and the disease was not curable.<br>*At that point I said no, no, he looks just like (name) and there's nothing wrong with him. I kind of went into denial...a denial mechanism set in. And then that evening we went out for a cup of coffee with the woman in the staff room and were...like...completely destroyed. In every way. So she talked...and we asked her questions, and...for better or for worse, at that time, I can see afterwards, she went through it all, and it was just hell on earth. It was such a shock. It was terrible, hearing the whole thing.*[1]<br>The first period after the diagnosis was made, was characterised by severe shock, anger and sadness; some mothers compared this period to a "tombstone", "the torture of Sisyphus" and "the end of happiness". The mourning for the loss of the "normal" child was apparent in the narratives of mothers, but it would be inappropriate to conclude that this is continuous or permanent. Mothers reported that they experience anxiety, concern, uncertainty, puzzlement, threat, shock, sadness, distress, anger and the diagnosis and differential diagnosis are presented as time-consuming and quite demanding procedures.[2]
Positive psychological impact<br>Positive psychological impact of labelling	In a similar manner, parents often described elation or relief when they received a diagnosis.[3]<br>…parents described their sense of relief when they finally got the diagnosis. At last they knew what was wrong with their child and their beliefs were confirmed that there was something more than just various symptoms and diseases, unrelated to each other.[1]
Mixed psychological impact<br>Both positive and negative impact of labelling	…ambivalence between relief and sorrow that illustrates the feelings of the parents when their child received the [diagnosis]. On the one hand the parents felt relieved that they finally found out what was wrong with their child, but on the other hand they felt sorrow because the diagnosis was final and not curable.[1]<br>The experience of the diagnosis announcement was reported by parents as an emotional "shock", a "relief" or "both a shock and a relief", regardless of the possible inheritance: *"It was a relief to know what she has got, but it was also a shock, because there is no solution, no way to repair it"*[4]
Psychological adaptation<br>Psychological adaptation to label and coping strategies/ mechanisms	Other parents actively resisted other people's negative labels, such as 'ill', 'sick' or 'disabled', finding their own language to describe their child's condition (e.g. 'different' or 'delayed') and restore their child's relationship to others:<br>*Even his sister doesn't know what's wrong with him… if I tell her, 'Oh, he's sick,' it will just put in her mind he is*

	sick, and he's not. He's learning from how he's playing with her, they are absolutely fine together. If she heard that from the other children, she will think he has a problem…He's different, yes. He is delayed, yes, but he's the same as the other children. (PPT 15)[5]<br>…the parents lowered their expectation of their child's abilities and level of current functioning, while at the same time not abandoning their hopes for progress that would open up other abilities in the future.[6]
Social identity<br>Changes to social identity as a result of label, including becoming a member/ mentor of a support group	Parent support groups were the most commonly reported coping resources; providing (emotional and practical) information and support: *... I found that [diagnosis] group a great help ... it was the first time I'd gotten a bit of positive feedback from somebody ... they were just SO nice and so honest and they were talking about their kids and I was telling them about [my son] ... they were just people like me ... I wasn't making excuses. I think when you meet people who are in similar situation as yourself you don't feel as crazy or as 'isolated'... I didn't feel like the odd one with the odd kid* (mother, son 13 years).[3]<br>…seeking to transmit to others what she has learned from her own experiences, says: *I created this website to help others.*[6]
Social stigma<br>Perceptions/ assumptions of others towards individual labelled	The isolation of the couple sprang in part from a reluctance to seek help from a social network deemed unable to comprehend the reality of the disease at such a young age as well as from the fear of stigma and bias.[7]<br>While the notions of inclusion and diversity are echoed in the mothers' discourse, the depiction of [diagnosis] and disability in the media seems to reinforce negative stereotypes. The vicious cycle includes the negative reactions of others towards children in the [diagnosis] which are partly due to their ignorance about [diagnosis] and which favour in turn the isolation of children in the [diagnosis] within the confines of their home.[2]
Medicalisation<br>Asymptomatic labels and understanding/ perception of symptoms	Two features of [diagnosis] were identified as particularly difficult to understand: the disruptive behaviour associated with [diagnosis] and the invisibility of the condition: *He doesn't come across different when we go out, so that hides it ...People don't see him [as] different, so people don't really understand. (PPT6)*[5]<br>…other participants expressed frustration about the recent popularisation (e.g. in the media) of using the [diagnosis] to describe any meticulous or ritualistic behaviour in a person without [diagnosis]. These participants believed that this misuse could result in the trivialisation of a debilitating mental health problem. Here, a more dichotomous view of illness and wellness was emphasised, such that [diagnosis] should only be applied to behaviour that causes dysfunction: *"...people say they've got [diagnosis] because they're very fussy and particular and they like their books in order and things like that. But I think that trivialises it because a lot of people say 'I'm very [diagnosis] about this, I'm [diagnosis] about that' but that's not an illness, I think it's only an illness when it becomes debilitating." (participant 7, wife)*[8]
**Support**	
Close relationships	Mothers appeared to set goals and take action and initiatives, approaching the roles of therapists, educators, special educators, psychologists, speech therapists and other specialists without however, substituting for the actual

Managing relationships and interactions; support required, offered, and accepted following labelling	professionals. Dealing with a child with [diagnosis] meant they had to go beyond the traditional maternal role of child rearing, to acquire more dimensions and to include specialised skills and interventions. Indeed, the skills developed by these mothers were not the usual skills found in mothers of children without [diagnosis]. The mothers studied books about [diagnosis], referred to websites of scientific associations and professionals, worked alongside their child's therapists, learned from each other, participated in seminars and conferences, and by developing a critical view of the interventions applied they managed to apply themselves to some elements of those interventions.[2]<br>However, participants witnessed in family members an emerging internal struggle with two views of self, that of 'relative' and that of 'carer.' *I think it's a real struggle to try and be the person who is the carer…first of all you're the wife…that's your relationship; second, you're taking on a role…*[9]<br>Similarly, carers witnessed 'a stronger family bond amongst the siblings' in many families as siblings once considered in a 'shambles' 'had to bond together' to coordinate support.[9]
Healthcare professionals interactions/ relationships<br>Interactions with healthcare professionals; support provided; explanations	Coming here helped. Many couples described feeling helped and supported by individual health professionals, despite their general dissatisfaction with the services offered. Couples perceived individual clinicians as caring and supportive but unable to really offer them practical help or advice.[10]<br>Carers/family often reported a lack of involvement and support from clinicians, including poor provision of information and limited opportunity for discussion, which could have negative, indirect influences on service user experience.[11]
Emotional support reduced/ limited<br>Emotional support lost as a result of label or support absent but perceived to be required	Alongside labelling and stereotyping, participants described the social separation that occurred as a result of their child's [diagnosis]. This separation, a hallmark of stigma, began with children being labelled sick, mentally ill, different or disabled, and led to their and their families' physical separation from others.<br>*[Other families] separate my son from the other children. Like, he [has] a problem. Like …'Don't go near the sick child.' It's not a good thing for my son. (PPT 15)*[5]<br>…the mothers discussed issues of acceptance, rejection, stigma and struggle against discrimination. Some mothers reported loneliness and isolation as a consequence of rejection by the social environment, an actual experience of social exclusion, and some reported loss of friends and social life.[2]
Emotional support increased/ maintained<br>Emotional support maintained or increased as a result of label	Instead of expressing rejection or fear of the patients, family members tended to emphasize the implications of [diagnosis], including fear of the loss of a loved one; the burden of care for the patient and, perhaps, children that are left behind after the patient's death; and loss of future plans.[12]<br>Coping very well. Couples described a process of finding strategies to help them cope with their current difficulties as a couple and as individuals, which included the support they were receiving from other people: *'That's how we cope with it, with help from others, otherwise it's difficult' [Wife]*[10]
Disclosure	Subjects often do not tell their neighbours that they have [diagnosis] because they perceive that stigma will result.[12]

Fear and methods of disclosing label to others (friends/family/ employers/colleagues)	Some couples also continued to minimise the permanence of the [symptoms] and the impact on their daily life. These couples began to isolate themselves from others, not wanting others to know about the diagnosis.[10]
**Future planning**	
Action<br>Forward planning and decision making as a result of label	…provided them with knowledge and possibilities to guide and support their daughter or son in different ways.[1]<br>Anna and Harry had wanted to have another baby but, due to the diagnosis, did not go on to do so.[13]
Uncertainty<br>Forward planning and decision making as a result of label	The uncertain future was also discussed by Molly and Jim. Molly quickly marked Ruby 'completely healthy', but Jim suggested it is less binary, *"[…] up towards this end"*. Despite her previous certainty, Molly acknowledged the "chance" of a less positive outcome: *"[The doctor] said, 'We still don't know what 30 years, 40 years will look like, on her lungs', so y'know it's still keeping that in in the back of our mind all the time that there is that chance […] Especially cos they don't know what the future could look like, it's that uncertainty now, for this type of generation."* Molly notes that [diagnosis] long-term prognosis is unknown for both parents and professionals, which again may shake the traditional view of medicine as a certain institution.[13]<br>The mothers were mostly concerned with the future prospects for their child with [diagnosis] and narrated their worries about their child's independence, adulthood, employment and the social effects of financial crisis on education, health care and disability.[2]
**Behaviour**	
Beneficial behaviour modifications<br>Behaviour modification/ changes as a result of labelling beneficial to overall health and wellbeing	*[…] whether there was kind of even a consideration as to whether people need to know if their child's got [diagnosis]? Erm, but I would say for us, I would still want to know—cos even though it's not impacting on our life we're still doing things as preventative, to make sure that she's gonna be as healthy as possible, even though she's not symptomatic. So there would never be an occasion where I think, 'oh I wish I didn't know'."* Jim's hesitance suggests he may have felt uncomfortable raising this, as medicine is traditionally revered. Jim concluded that knowing about [diagnosis] is right for his family but acknowledged that others may feel differently. Interestingly, a healthy child in context of [diagnosis] becomes "not symptomatic", and routine healthy choices become "preventative".[13]
Detrimental/ unhelpful behaviour modifications<br>Behaviour modification/ changes as a result of labelling unhelpful/	Sue appears to need to hyper-control the environment, suggesting that disease is a constant threat. Similarly, Molly remained anxious that 4-year-old Ruby's activities were potentially *"dangerous for your lungs"* (Molly).[13]<br>Some couples also continued to minimise the permanence of the memory problems and the impact on their daily life. These couples began to isolate themselves from others, not wanting others to know about the diagnosis.[10]

restrictive to overall health and wellbeing	
**Treatment expectations**	
Positive treatment experiences Perceptions of treatment/ intervention (and outcomes) to be positive/ beneficial	Parents reported positive perceptions and experiences with medication. For example, one mother (son 13 years) said *'... it was like a puzzle falling into place ... it was like great we found something that works ...'.*[3]
Negative treatment experiences Perceptions of treatment/ intervention (and outcomes) to be negative/ unhelpful	Limited availability and accessibility and lack of flexibility and continuity were the faults cited by the spouses in reference to the home care, psychological support, and respite services that they needed: *I really tried to find someplace where I could be alone ... but we have a child. In my case, if I ask this service to come here, well I have to get my son out of the house, which means that this service is for someone with no children ... or for an elderly couple. (Interview 05—female, age 46, caring for spouse with [diagnosis]).*[7] All the mothers reported using costly private services for their [diagnosis] children, which included interventions by specialists after school either by individuals at home or in organised intervention centres. This is a financial burden for all parents because the Greek National Insurance Service covers only a small amount of the expenses.[2]

2.2 Supplementary Table 2 Major and subthemes arising as consequences for the healthcare professionals

**Theme,** *Subtheme*, Description	**Exemplary comment**
**Psychosocial impact**	
Negative psychological impact<br>Negative psychological impact of labelling	When participants expressed hesitation about disclosing the diagnosis to their patients, their language evoked negative connotations such as "bad news" and "stigma", and they expressed concerns about the potentially negative psychological impact of disclosure on patients.[14]<br>Some GPs and practice nurses stated that they avoided the diagnosis as they believed that there was no cure for [diagnosis] or that the label may be harmful and act to exacerbate the symptoms.[15]
Positive psychological impact<br>Positive psychological impact of labelling	Some GPs believed that the label of [diagnosis] can be helpful for the patient in giving a name to their symptoms: "*Some people like a label, some people like to know what's causing their symptoms whether it's the truth or not and some people are looking for a label to attach to their symptoms." (GP17)*[16]<br>However, [diagnosis] specialists and other GPs did recognise the importance of a positively framed diagnosis, and the impact this can have on the patient's quality of life: *"Actually making a diagnosis can be quite empowering for patients as long as all of the causes have been excluded, that all red flags have been excluded, et cetera, and that the clinical history sort of makes sense, the story, listening to the patient's story, when you've heard it many times it seems fairly obvious but you actually need to make time to hear it." [diagnosis] specialist 2.*[15]
Mixed psychological impact<br>Both positive and negative impact of labelling	However, this value was generally considered to be limited and short-lived: *"At a superficial level it's empowering because it gives them control over their life and their work, but at a deeper level it prevents them from engaging fully with the existential conditions of their life which is what they can't cope with." (GP18)*[16]<br>Some felt that although the diagnosis carried some stigma, it was still important to know as it gave women 'a clear way forward'.[17]
Psychological adaptation<br>Psychological adaptation to label and coping strategies/ mechanisms	coping response may have the potential to evolve into avoidance. This could lead to some patients to become isolated and refuse to accept any sort of intervention, even when HCPs approach them directly: "*There's...well, patients that go completely off the radar and won't answer the phone. And they are completely uncontactable." FG1*[18]
Self-Identity<br>Changes to self-identity following provision of label (can be positive or negative)	…the benefits of social inclusion were endorsed by the majority of clinicians. Increasing interaction with peers was seen as an effective method of ensuring young people felt 'normal' as well as of actively challenging heightened feelings of suspiciousness and social isolation.[19]<br>Participants stressed that especially when dealing with a person in the first stages of the disease, the experts' diagnostic label required by the courts might be problematic and might even have vicious consequences, especially when it comes to respecting the autonomy of individuals with [diagnosis].<br>*Experts' certifications might be a problem. They label the person as having [diagnosis]… Especially in the first*

	stages of the disease, this might be detrimental. Even if afterwards we try to protect his rights, the label is very damaging. (A2, SW, FG2)[20]
Social stigma<br>Perceptions/ assumptions of others towards individual labelled	Potential stigma associated with PCOS: *"If the perception is that a [diagnosis] is related to being obese and having poor lifestyle behaviours, then if you tell someone who is slender and fit that they have [diagnosis] then they sort of feel that that's a stigma. It's sort of a slight on their perception of their health.' (ID20, Gyn, practicing 16 years)*[17]<br>Some psychiatrists thought that stigmatisation could never be completely eliminated because of the nature of certain types of mental illness, especially [diagnosis]. There were some typical symptoms and behaviours presented by the patients.<br>*For example let's say that psychiatric patients are not violent. This is not entirely correct because some of the [diagnosis] patients are violent. As this is the real situation in some patients, zero stigmatisation is never going to happen. For example, [diagnosis]. Although most of the [diagnosis] patients will not infect other people, some will. This minority leads all [diagnosis] patients to suffer from stigmatisation. It is the same with mental disorder. Some mental illnesses are characterised with violence, eccentricity, or deviance. These aberrant characteristics make people doubt if the illness is curable. (GP2, P1)*[21]
Medicalisation<br>Asymptomatic label and understanding/ perception of symptoms	Participants in the focus groups felt that the medical label of [diagnosis] was often a quick fix that stopped other questions being asked about what else might be going on. *"Much easier for a parent to feel my baby has a medical cause than maybe I'm not coping. Much easier for a doctor to say it's [diagnosis], I can do something about that but I don't have time to spend an hour asking why your relationship with your mother is so poor that you're not coping and you've got a past history of attachment disorder. So I think it comes both from doctor, I think it comes from expectation of parent, there's media, there's hype, there's a lot of stuff out there about crying babies. You type in crying baby, you see [diagnosis]." (*Paediatrician).[22]<br>All six clinicians reported difficulty in determining which behaviours were aspects of normal adolescent development and which were suggestive of an emerging [diagnosis]: *"Is it teenage [symptom] from associated stressful situations or low mood such as traumatic experiences that have resulted in someone becoming sort of suicidal or self harming? They [young people] are maybe talking about voice experiences when actually...it is more internalised thoughts and confusion" (PP1, 27).*[19]
**Support**	
Close relationships<br>Managing relationships and interactions; support required, offered, and accepted following labelling	Also observed that family members often assumed a significant role in the help-seeking process and made decisions on behalf of the patients[21]<br>The degree of [symptoms] suffered by the patient and the need to act in the patients' best interests appeared to guide thinking: *'One of the difficulties... in the early stages is the issues of communicating back to family and carers about someone who is legally competent ... I've got to say to the patient... "We should really talk to the family about this and bring them in with you" ... And that's often quite a sticky time.'(GP14)*[23]

Healthcare professionals interactions/ relationships<br>Interactions with healthcare professionals; support provided; explanations	The desire to avoid unnecessary anxiety or harm was mentioned by most clinicians as a reason for not informing some older patients or for "down playing" the impact of [diagnosis].<br>*"So I suppose I think patients do have a right to know about their health when it's going to affect their health, but if you weigh it all up as a doctor and you think actually, this is going to cause more harm than help, because this is a 95 year old that's really anxious and already struggling with some other medical problems ... then you've got to think really, why would you tell that person?" GP 11*[24]<br>Other clinicians, particularly those concerned about the negative impact of disease labelling, described avoiding the label entirely and focusing on treating bothersome symptoms and encouraging a healthy lifestyle. This strategy, however, is not always successful if patients (or their parents in the case of adolescents) insist on a diagnosis.<br>*'We just have to be very cautious in labelling them with conditions that are going to stay with them for a long time if there's any level of uncertainty.' (ID30, Endo, practicing 30 years)*[17]
Emotional support increased/ maintained<br>Emotional support maintained or increased as a result of label	The role of supporting the patient was stressed by respondents: *'I think one of the crucial things for these kinds of people is for a doctor to say "I'm on your side, I'm going to be with you through thick and thin", and for the doctor to accept their relative powerlessness, but none the less to accompany the patient through this.' (GP17)*[16]<br>These participants described families reaching beyond embarrassed shame and unrequited hope to extend relational opportunities to other [diagnosis] families.[9]
Disclosure<br>Fear and methods of disclosing label to others (friends/family/ employers/colleagues)	Disclosure was *'easier if the patient is thinking about that diagnosis'*[23]<br>The secrecy of families 'embarrassed to tell' because others 'don't understand what [diagnosis] is' was recognised by these senior carers as part of the overarching stigma of aged care, perpetuated through 'helplessness,' shame, 'fear,' and naivety.[9]
Secondary gain<br>Gains from label	In some of the areas, practices were being financially incentivised to diagnose patients with [diagnosis] and/or refer onto the [treatment program]. In two of the sampled practices, automated systems were put into place as a direct response to the financial incentivisation of [diagnosis] case finding. The comment below came from a GP in an area where a local scheme exists that pays practices for each patient they diagnose as [diagnosis] who has a documented BMI and receives lifestyle change advice.<br>*"[when discussing why the clinicians in the group would still identify and assess a 95-year-old patient with [diagnosis] in a nursing home] Yeah, rather than what the patient's age is, we've got to show that we are identifying these results, we are providing the health education and doing the relevant health checks for these patients." GP in focus group 2*[24]<br>But there was also a sense in which some subjects construed patients as seeking a more explicit personal gain. *"What else is going on? That's the question that springs to mind. [diagnosis] is the new back pain, you know. I don't think people look hard enough at the secondary gains of illness. I think particularly now the government is willing us back towards full employment, the only way out of working for your living is to be ill." [GP17 (1)]*[25]

**Future planning**	
Action<br>Forward planning and decision making as a result of label	For many clinicians, particularly GPs, a diagnosis is important because it starts discussions about optimal health and facilitates a holistic, collective approach to symptom management.[17]<br>A diagnosis of [diagnosis] has *'implications for the patient and the family' (GP15) 'and most...carers actually really...want to know what they can do about it'*[23]
**Behaviour**	
Beneficial behaviour modifications<br>Behaviour modification/ changes as a result of labelling beneficial to overall health and wellbeing	A few also discussed how the diagnosis enables lifestyle to be addressed in a non-stigmatising way.[17]<br>When considering the psychological impact of a [diagnosis], several HCPs identified the need for patients to retain control over nutritional decision-making. This was viewed as a potentially empowering factor for patients and consisted of the possibility to retain decision-making and control within the context of nutrition: *I think supporting them to still be able to have nutrition is a key thing, in sort of empowering them, to still be able to have ownership of that. FG2* [18]
Detrimental/ unhelpful behaviour modifications<br>Behaviour modification/ changes as a result of labelling unhelpful/ restrictive to overall health and wellbeing	These clinicians perceived that 'over-medicalising' and labelling weight issues as [diagnosis] undermines patients' sense of 'agency' and control over their weight, while recognising patients' preference for a medical explanation of their obesity (ID35, Endo, practicing 28 years).<br>*'I'm not sure that we do people any favours by giving them a label. You might be also setting them up for failure by giving them a label to something that they may not necessarily have and then the treatments probably won't necessarily help their situation.' (D34, Endo, practicing 15 years*[17]<br>The negative and marked difference between 'being labeled' and 'not being labeled' within the legal system was further explained by A2, a municipal social welfare officer: *I mean, a person can have [symptoms], but no diagnosis. So, he/she will not be labeled as such; he/she can still be part of society, the family will respect him/her, and he/she will be able to perform all the legal actions he/she needs to take. But once he/she's been labeled as having [diagnosis] as a result of a diagnostic certificate provided by a physician, everything will change for him/her. (A2, SW, FG2)*[20]
**Treatment expectations**	
Positive treatment experiences<br>Perceptions of treatment/ intervention (and outcomes) to be positive/ beneficial	This gain also allows the GP to follow a pre-determined treatment plan: *"With the good sides of [medications], it helps us to stagger the consultations, being able to prescribe and review somebody 2 to 3 weeks later, and again 2 to 3 weeks later, is a good way of breaking up those consultations we don't have time for, it makes us feel good because it feels as if we are doing something, it makes us feel good because we know that the patient will improve if we have got the diagnosis right and they take the tablets." [GP2 (2)]*[25]

	They explained that there was a need to clinically classify the patients according to their diagnoses based on which the treatment plan was delineated.[21]
Negative treatment experiences<br>Perceptions of treatment/ intervention (and outcomes) to be negative/ unhelpful	GPs reported, therefore, that the limited resources available in both primary and secondary care forced them to prescribe [medications] rather than psychological therapies:<br>*"It takes forever to get patients to be seen. If you refer someone who is depressed it could take 4–6months before they get an appointment . . . Erm, nothing much happens when they get there, funnily enough . . . they change the antidepressant and see how they feel in a few months—well, I could have done that, you know." [GP9 (1)]*[25]<br>In many cases clinicians described how there was a lack of consistency, agreement and uncertainty within teams and between services in terms of how to work with individuals identified as having an [diagnosis]:<br>*'I think it would be nice for everyone to be kind of singing off the same kind of hymn sheet really ...knowing exactly what an [diagnosis] is...and maybes just some kind of training...might standardise it so that everyone knows exactly what the definition is, how to assess, how to manage and how to treat people that present with an [diagnosis] (PP4, 23).'*[19]

2.3 Supplementary Table 3 Major and subthemes arising as consequences for the community

**Theme,** *Subtheme*	**Exemplary comment**
**Psychosocial impact**	
Social identity<br>Changes to social identity as a result of label, including becoming a member/ mentor of a support group	Some participants thought that the attitudes and behaviours of health staff towards patients with [diagnosis], especially the way they relate to, and treat those with [diagnosis] make the disease shameful.[26]<br>According to the participants, the judges had great difficulties understanding that in the case of a person with [diagnosis], the diagnostic label is not associated with a total loss of competence and that, indeed, a person with [diagnosis] might retain the ability to perform some activities of daily living, while still not being able to make decisions for himself/herself in other areas.[20]
Social stigma<br>Perceptions/ assumptions of others towards individual labelled	Community leaders described how people with [diagnosis] could be given stigmatizing labels such as 'lazy', 'liars', or 'crazy' by their community and [Black and Minority Ethnicity] patients may therefore want to avoid this potentially stigmatizing diagnosis.[27]
**Support**	
Emotional support reduced/ limited<br>Emotional support lost as a result of label or support absent but perceived to be required	Some of the participants described witnessing even stronger reactions by the courts when dealing with a case involving a person with a confirmed diagnosis of [diagnosis]: *In these cases (a diagnostic label of [diagnosis]) they (the judges) want to get rid of the case. (M2, Lawyer, FG2)*[20]
Emotional support increased/ maintained<br>Emotional support maintained or increased as a result of label	Lawyers stressed the importance of using diagnostic labels to prove the individual's vulnerable status, and as a signal that this person needs protection from the courts. *I think that this (having a diagnostic label) serves as a reference that the person indeed has [diagnosis] and that the legal system must protect him. (M2, Lawyer, FG2)*[20]
**Behaviour**	
Detrimental/ unhelpful behaviour modifications<br>Behaviour modification/ changes as a result of	However, in the case of granting guardianship, the diagnostic label was perceived as leading to negative consequences, such as ignoring the person with [diagnosis], infringing on his/her human rights, personal preferences and ability to make autonomous decisions. As reflected in the words of one of the participants: ... *Indeed, there are two [legal] paths: One path in which you are trying hard to demonstrate the person is sick to get all sorts of (rights*

labelling unhelpful/ restrictive	*and benefits), insurances, and so on; and many other times one in which we try to demonstrate that, despite having [diagnosis] the individual has abilities and there is no need to appoint a guardian. (FG1)*[20]
**Treatment expectations**	
Negative treatment experiences Perceptions of treatment/ intervention (and outcomes) to be negative/ unhelpful	…less likely to suggest a diagnosis of [diagnosis], and this can contribute to patients not seeking a medical opinion. *"…The doctor just says 'oh look after him', and all that, you, know, and not really referring them on to the hospital to be diagnosed properly" Community Leader 3, male, Indian*[27]

2.4 References associated with quotes provided in Supplementary Tables 1-3

1. Hallberg U, Óskarsdóttir S, Klingberg G. 22q11 deletion syndrome - the meaning of a diagnosis. A qualitative study on parental perspectives. *Child Care Health Dev* 2010;36:719-725. doi:10.1111/j.1365-2214.2010.01108.x.
2. Loukisas TD, Papoudi D. Mothers' Experiences of Children in the Autistic Spectrum in Greece: Narratives of Development, Education and Disability Across their Blogs. *Int J Disabil Dev Educ* 2016;63:64-78. doi:10.1080/1034912X.2015.1111304.
3. Carr-Fanning K, McGuckin C. The powerless or the empowered? Stakeholders' experiences of diagnosis and treatment for attention-deficit hyperactivity disorder in Ireland. *Ir J Psychol Med* 2018;35:203-212. doi:10.1017/ipm.2018.13.
4. Houdayer F, Gargiulo M, Frischmann M, et al. The psychological impact of cryptic chromosomal abnormalities diagnosis announcement. *Eur J Med Genet* 2013;56:585-90. doi:10.1016/j.ejmg.2013.09.002.
5. Selman EL, Fox F, Aabe N, Turner K, Rai D, Redwood S. 'You are labelled by your children's disability' - a community-based, participatory study of stigma among Somali parents of children with autism living in the United Kingdom. *Ethn Health* 2018;23:781-796. doi:10.1080/13557858.2017.1294663.
6. Fleischmann A. The hero's story and autism: grounded theory study of websites for parents of children with autism. *Autism* 2005;9:299-316. doi:10.1177/1362361305054410.
7. Ducharme F, Kergoat M-J, Antoine P, Pasquier F, Coulombe R. The unique experience of spouses in early-onset dementia. *Am J Alzheimers Dis Other Demen* 2013;28:634-641. doi:10.1177/1533317513494443.
8. Pedley R, Bee P, Berry K, Wearden A. Separating obsessive-compulsive disorder from the self. A qualitative study of family member perceptions. *BMC Psychiatry* 2017;17:326. doi:10.1186/s12888-017-1470-4.
9. Walmsley B, McCormack L. Shame, hope, intimacy and growth: dementia distress and growth in families from the perspective of senior aged care professionals. *Demen* 2016;15:1666-1684. doi:10.1177/1471301215573676.
10. Robinson L, Clare L, Evans K. Making sense of dementia and adjusting to loss: psychological reactions to a diagnosis of dementia in couples. *Aging Ment Health* 2005;9:337-47. doi:10.1080/13607860500114555.
11. Perkins A, Ridler J, Browes D, Peryer G, Notley C, Hackmann C. Experiencing mental health diagnosis: a systematic review of service user, clinician, and carer perspectives across clinical settings. *Lancet Psychiatry* 2018;5:747-764. doi:10.1016/s2215-0366(18)30095-6.
12. McGrath JW, Ankrah EM, Schumann DA, Nkumbi S, Lubega M. AIDS and the urban family: its impact in Kampala, Uganda. *AIDS Care* 1993;5:55-70. doi:10.1080/09540129308258584.
13. Johnson F, Southern K, W, Ulph F. Psychological Impact on parents of an inconclusive diagnosis following newborn bloodspot screening for cystic fibrosis: a qualitative study. *Int J Neonatal Screen* 2019;5:23. doi:10.3390/ijns5020023.
14. Abe M, Tsunawaki S, Matsuda M, Cigolles CT, Fetters MD, Inoue M. Perspectives on disclosure of the dementia diagnosis among primary care physicians in Japan: a qualitatively driven mixed methods study. *BMC Fam Pract* 2019;2069. doi:10.1186/s12875-019-0964-1.
15. Hannon K, Peters S, Fisher L, et al. Developing resources to support the diagnosis and management of chronic fatigue syndrome/myalgic encephalitis (CFS/ME) in primary care: a qualitative study. *BMC Fam Pract* 2012;13:93. doi:10.1186/1471-2296-13-93.

16. Chew-Graham C, Dowrick C, Wearden A, Richardson V, Peters S. Making the diagnosis of chronic fatigue syndrome/myalgic encephalitis in primary care: a qualitative study. *BMC Fam Pract* 2010;1116. doi:10.1186/1471-2296-11-16.
17. Copp T, Muscat DM, Hersch J, et al. Clinicians' perspectives on diagnosing polycystic ovary syndrome in Australia: a qualitative study. *Hum Reprod* 2020;35:660-668. doi:10.1093/humrep/deaa005.
18. Zarotti N, Coates E, McGeachan A, et al. Health care professionals' views on psychological factors affecting nutritional behaviour in people with motor neuron disease: a thematic analysis. *Br J Health Psychol* 2019;24:953-969. doi:10.1111/bjhp.12388.
19. Welsh P, Tiffin PA. Experience of child and adolescent mental health clinicians working within an at-risk mental state for psychosis service: a qualitative study. *Early Interv Psychiatry* 2012;6:207-211. doi:10.1111/j.1751-7893.2012.00352.x.
20. Werner P, Doron, II. Alzheimer's disease and the law: positive and negative consequences of structural stigma and labeling in the legal system. *Aging Ment Health* 2017;21:1206-1213. doi:10.1080/13607863.2016.1211989.
21. Sun KS, Lam TP, Lo TL, Wu D. How Chinese psychiatrists see and manage stigmatisation of psychiatric patients: a qualitative study in Hong Kong. *Evid Based Ment Health* 2019;22:51-55. doi:10.1136/ebmental-2018-300078.
22. Dahlen HG, Foster JP, Psaila K, et al. Gastro-oesophageal reflux: a mixed methods study of infants admitted to hospital in the first 12 months following birth in NSW (2000-2011). *BMC Pediatrics* 2018;18:1-1. doi:10.1186/s12887-018-0999-9.
23. Phillips J, Pond CD, Paterson NE, et al. Difficulties in disclosing the diagnosis of dementia: a qualitative study in general practice. *Br J Gen Pract* 2012;62:e546-53. doi:10.3399/bjgp12X653598.
24. Burch P, Blakeman T, Bower P, Sanders C. Understanding the diagnosis of pre-diabetes in patients aged over 85 in English primary care: a qualitative study. *BMC Fam Pract* 2019;20:90. doi:10.1186/s12875-019-0981-0.
25. Chew-Graham CA, Mullin S, May CR, Hedley S, Cole H. Managing depression in primary care: another example of the inverse care law? *Fam Pract* 2002;19:632-7. doi:10.1093/fampra/19.6.632.
26. Dodor EA, Kelly S, Neal K. Health professionals as stigmatisers of tuberculosis: insights from community members and patients with TB in an urban district in Ghana. *Psychol Health Med* 2009;14:301-10. doi:10.1080/13548500902730127.
27. De Silva RE, Bayliss K, Riste L, Chew-Graham CA. Diagnosing chronic fatigue syndrome in South Asians: lessons from a secondary analysis of a UK qualitative study. *J Fam Med Prim Care* 2013;2:277-82. doi:10.4103/2249-4863.120765.
